# Hyperpathway: visualizing organization of pathway-molecule enriched interactions in omics studies via hyperbolic bipartite network embedding

**DOI:** 10.1038/s41540-026-00752-w

**Published:** 2026-06-09

**Authors:** Ilyes Abdelhamid, Ziheng Liao, Yuchi Liu, Armel Lefebvre, Aldo Acevedo, Carlo Vittorio Cannistraci

**Affiliations:** 1 Center for Complex Network Intelligence (CCNI), Tsinghua Laboratory of Brain and Intelligence (THBI), Department of Psychological and Cognitive Sciences, Tsinghua University, Beijing, China; 2 School of Biomedical Engineering, Tsinghua University, Beijing, China; 3 Department of Computer Science, Tsinghua University, Beijing, China; 4 Health Campus The Hague, Department of Public Health, Primary Care, Leiden University Medical Center, The Hague, The Netherlands; 5 Leiden Institute of Advanced Computer Science (LIACS), Leiden University, Leiden, The Netherlands; 6 Formerly at Biomedical Cybernetics Group, Biotechnology Center (BIOTEC), Center for Molecular and Cellular Bioengineering (CMCB), Technische Universität Dresden, Dresden, Germany

**Keywords:** Computational biology and bioinformatics, Systems biology

## Abstract

Pathway enrichment analysis (PEA) of omics data identifies significant pathway–molecule associations, yet delivers results as tabular lists in which complex systems-biology insights remain inaccessible. Hyperpathway is an open-access network-based webtool that addresses this limitation through three original innovations: (1) conversion of a PEA results table into a pathway–molecule bipartite network; (2) a minimal artificial linking strategy to resolve structural disconnections; (3) a leaf removal and post-hoc reinsertion pipeline that accelerates coalescent embedding without any loss of geometric fidelity. The resulting network is visualized in a two-dimensional hyperbolic disk with flexible coloring schemes encoding hierarchical relevance, connectivity similarity, statistical significance, or user-defined annotations; revealing latent functional modules that are invisible in conventional tabular outputs. Validated on genomic, metabolomic, and lipidomic datasets, Hyperpathway enables a deeper, systems-level understanding of the interplay between pathways and their molecular components, providing insights that go beyond p-value-based significance testing. Beyond PEA, Hyperpathway can be used as a general-purpose open webtool for fast hyperbolic embedding and interactive visualization of any bipartite network.

## Introduction

Pathway enrichment analysis (PEA) is a pivotal method in systems biology to uncover the functional implications of biological data by interpreting the role of a set of molecules—often discriminative for a certain phenotype—within the context of predefined biological pathways^1^. The pathways are molecular circuits that implement a biological function: they are characterized by networks of genes, lipids, metabolites, and other molecules involved in critical biological processes. In biomedical omics studies, particularly in genomics and metabolomics, researchers aim to identify molecular signatures—sets of genes, metabolites, or other biomolecules - that exhibit significant changes under specific biological conditions^2,3^. However, interpreting these signatures requires a secondary analysis step to contextualize them within biological processes and identify relevant functional pathways^4^. Traditional PEA approaches, such as Over Representation Analysis (ORA)^5^ and Functional Class Scoring (FCS)^6^, have become widely used to identify biologically relevant pathways for molecular signatures learned from high-throughput biological data^7,8^. However, these methods have inherent limitations, particularly in how they treat pathway components and interactions. While ORA relies on statistical significance to detect enriched pathways, it often disregards the relationships between pathway components, leading to oversimplified interpretations^9,10^. FCS methods, on the other hand, extend ORA by considering coordinated changes in gene expression, offering a more nuanced approach but still treating pathways as static gene sets, failing to capture the interplays between pathway components^6,11^.

To address the increasing scale and complexity of omics data interpretation, numerous functional enrichment tools have been developed across diverse domains, including GSEA for genomics^6^, MPEA and MBRole for metabolomics^4,12^, GeneTrail2 for multi-omics analysis^13^, and LIPEA for lipidomics^14^. While these tools excel at statistical enrichment detection, they predominantly present results as ranked tabular lists rather than network visualizations. Network-based visualization tools have emerged to enhance interpretability of PEA results such as ClueGO^15^ and Metascape^16^. However, these methods primarily visualize pathway–pathway relationships derived from shared molecules, rather than explicitly representing the bipartite network structure that reflects the full organization of pathways and their molecular components.

Hyperbolic geometry offers a natural framework to address this gap. Hyperbolic representations have recently been successfully applied in diverse fields, including, to name a few: Internet topology analysis^17^, social network community structure representation^18^, human brain connectome visualization and analysis^19,20^, protein–protein interaction mapping^21^, sparse artificial neural network architecture visualization and analysis^22^; demonstrating their ability to efficiently capture hierarchical and community-like patterns in large, heterogeneous datasets. In hyperbolic space, the radial coordinate of a node reflects its intrinsic centrality (a.k.a. popularity)^23^: nodes with higher degree are positioned closer to the center of the disk; while nodes with fewer connections are placed toward the periphery. Angular coordinates instead encode similarity: nodes that share similar interaction patterns are placed closer in angle. Together, radial and angular coordinates capture the hierarchical and similarity-based organization of the network, informed by prior studies showing that these properties emerge naturally in hyperbolic embeddings of molecular interaction systems^21,23,24^. These advances motivate extending hyperbolic network embedding to the biomedical domain, enabling the intuitive exploration of pathway–molecule relationships in omics data through a mathematically grounded geometric lens.

**Fig. 1:**
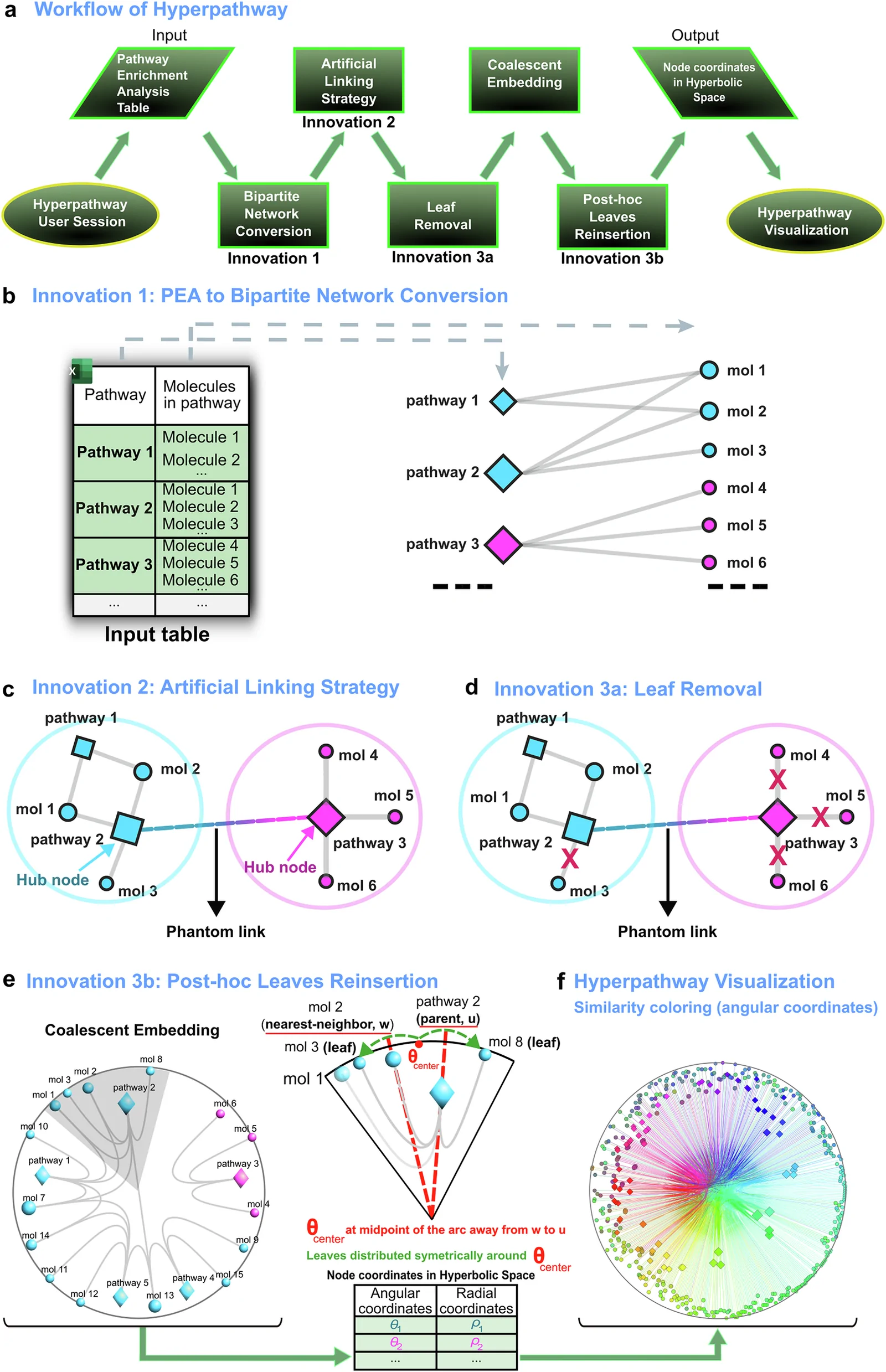
Hyperpathway: a computational framework for visualizing the hierarchical and modular organization of pathway–molecule interactions in hyperbolic space. **a** End-to-end pipeline: a Pathway Enrichment Analysis (PEA) table is processed through five successive steps—bipartite network conversion, artificial linking, leaf removal, coalescent hyperbolic embedding, and post-hoc leaf reinsertion—to produce node coordinates in hyperbolic space for visualization. **b** Innovation 1—PEA-to-bipartite network conversion. The input enrichment table is converted into a bipartite network in which diamond nodes represent pathways and circle nodes represent molecules, with edges encoding membership. **c** Innovation 2–Artificial Linking Strategy. If disconnected network components are present, they are mutually bridged via phantom links established between the hub nodes of each component, ensuring a single connected graph required for coalescent embedding. **d** Innovation 3a–Leaf Removal. Degree-1 nodes (leaves) are removed prior to embedding, reducing the distance matrix from $N\times {N}$ to ${N}_{{core}}\times {N}_{{core}}$ and thereby decreasing computational complexity. **e** Innovation 3b–Post-hoc Leaf Reinsertion. Following coalescent embedding of the core graph (left), pruned leaf nodes are reinserted post-hoc using a deterministic angular placement rule (right). For each leaf, the angular center ${\theta }_{{center}}$ is placed at the midpoint of the arc directed away from the nearest neighbor w in the RAA angular space, computed relative to the parent node u. When multiple leaves share the same parent, they are distributed symmetrically as a fan centered at ${\theta }_{{center}}$, with angular spacing equal to the equidistant step of the EA embedding ($\frac{2\pi }{{N}_{{core}}}$, where ${N}_{{core}}$ is the number of nodes in the core network). Radial coordinates for all nodes are assigned globally by fitting a power-law to the degree sequence of the full network, placing leaves naturally at the periphery of the hyperbolic disk. The resulting node coordinates (angular θ and radial ρ) for all nodes are reported in the output table, downloadable on the webtool. **f** Hyperpathway Visualization. Final embedding is displayed in the hyperbolic disc with similarity-based coloring by default, where angular proximity reflects functional relatedness between pathways and molecules; and radial coordinates their hierarchical position.

Here, we introduce Hyperpathway, a network-based computational framework (see Fig. 1a) for the visualization and interpretation of PEA results derived from omics studies; offering flexible visual differentiation of pathways and molecules by statistical significance, network geometry, and user-defined attributes (See “Methods”—Visualization of the embedded network in the 2D native hyperbolic disk). Our first innovation is (1) PEA-to-bipartite network conversion, where a PEA results table reporting statistically significant pathways and their enriched molecules is transformed into a formal pathway-molecule bipartite network (see Fig. 1b). We then address a key structural constraint of coalescent embedding by introducing (2) an artificial linking strategy, where disconnected components are minimally bridged to ensure a single connected graph without altering the intrinsic bipartite organization of the data (see Fig. 1c). To reduce computational complexity and enable embedding at scale, we implement (3a) leaf removal prior to embedding as degree-one nodes are geometrically invariant and are pruned, yielding a faster embedding of the core network (see Fig. 1d).

This core network is then embedded into a two-dimensional hyperbolic disk using coalescent embedding^18^, a widely recognized model-free methodology for mapping complex networks into the hyperbolic space (see Fig. 1e). Finally, we introduce (3b) a post-hoc leaf reinsertion (see Fig. 1e), which restores all pruned peripheral nodes into the finalized layout (see Fig. 1f) using local angular rules, ensuring a complete representation of the original network. We further validate the practical utility of this accelerated embedding workflow by benchmarking the computational speedup of the leaf removal and reinsertion pipeline against direct full-network embedding (see Fig. 5).

We demonstrate the utility of Hyperpathway by analyzing three public datasets encompassing genes, metabolites, and lipids. Our results reveal that representing pathway-molecule interactions in a hyperbolic bipartite network offers a more nuanced interpretation of biological information than traditional PEA alone. These results highlight the ability of Hyperpathway to identify central elements within biological networks associated with significant pathways, providing insights that go beyond p-value-based significance testing and therefore offering a robust add-on to traditional pathway enrichment methods. By bridging statistical enrichment analysis with the latent geometry of biological networks, Hyperpathway advances pathway analysis from a list-based to a systems-level geometric visualization of omics data. Beyond PEA, Hyperpathway also serves as a general-purpose, open web tool for rapid hyperbolic embedding and interactive visualization of arbitrary bipartite networks.

## Results

### Hyperpathway

Given a set of molecules significantly associated with a phenotype (e.g., a disease or a cellular state change), also known as a molecular signature, the standard PEA output is typically presented as a table (Fig. 1b). Each row reports: (i) a pathway, (ii) the subset of signature molecules (statistically) enriched in that pathway. Additional optional columns (iii–end) may include enrichment statistics, such as uncorrected and multiple-testing–adjusted *p*-values (e.g., Benjamini-Hochberg or Bonferroni correction), as well as user-defined attributes (e.g., pathway community membership; see Supplementary Fig. 1 and Supplementary Fig. 15). The first two columns define a bipartite structure linking pathways to molecules, whereas the additional columns (iii–end), including enrichment statistics and user-defined attributes, are subsequently used as visual annotations to encode statistical significance and contextual biological information in the final embedding. This bipartite structure arises because pathways connect only indirectly through shared enriched molecules, and molecules connect only indirectly by appearing in multiple enriched pathways. Such two-step indirect connections between nodes of the same class are a defining structural feature of bipartite networks^25–27^.

The first innovation of Hyperpathway is to convert a tabular representation of bipartite knowledge into a bipartite network of pathway-molecule interactions (see Fig. 1b), where diamond-shaped nodes represent pathways, circle-shaped nodes represent molecules, and edges encode membership. As real-world PEA outputs frequently yield disconnected graphs, due to molecules enriched in only a single pathway or pathways sharing no common molecules, Innovation 2 introduces an artificial linking strategy (see Fig. 1c). Disconnected components are minimally bridged via phantom links established between the highest-degree hub nodes of each component, ensuring a single connected graph required for coalescent embedding without altering the intrinsic bipartite organization of the data (see Methods – Innovation 2).

To enable efficient embedding at scale, Innovation 3a introduces a leaf removal step prior to embedding (Fig. 1d). Degree-one nodes (molecules connected to only one pathway, or pathways connected to only one molecule) are geometrically invariant: they do not influence the latent geometry of the network and their removal does not alter the hyperbolic coordinates of any remaining node. Pruning them reduces the distance matrix from N × N to ${N}_{{core}}$ × ${N}_{{core}}$, substantially decreasing the computational complexity of the embedding step (see Methods – Innovation 3a).

The resulting core network is then embedded into a two-dimensional hyperbolic disk using coalescent embedding, a model-free methodology for mapping complex networks into hyperbolic space (see “Methods”—Coalescent embedding of the PEA bipartite network). Following embedding, Innovation 3b restores all pruned leaf nodes into the finalized layout via a post-hoc reinsertion procedure (Fig. 1e): each leaf is placed at the periphery of the hyperbolic disk using a deterministic angular rule anchored to its parent node and to the parent’s nearest neighbor in the RAA angular space, ensuring a complete and topologically faithful representation of the original network *G* without modifying any core-node coordinate (see Methods – Innovation 3b). The combined leaf removal and reinsertion pipeline achieves a computational speedup of ×3.26 on a laptop (16 GB RAM) and ×3.03 on a workstation (256 GB RAM) relative to direct full-network embedding, as benchmarked on the full Reactome Pathways Gene Set (Fig. 5a). Web-based rendering of the final visualization remains interactive within 20 seconds for up to 50 pathways, with local execution consistently faster across all tested scales (Fig. 5b).

**Fig. 2:**
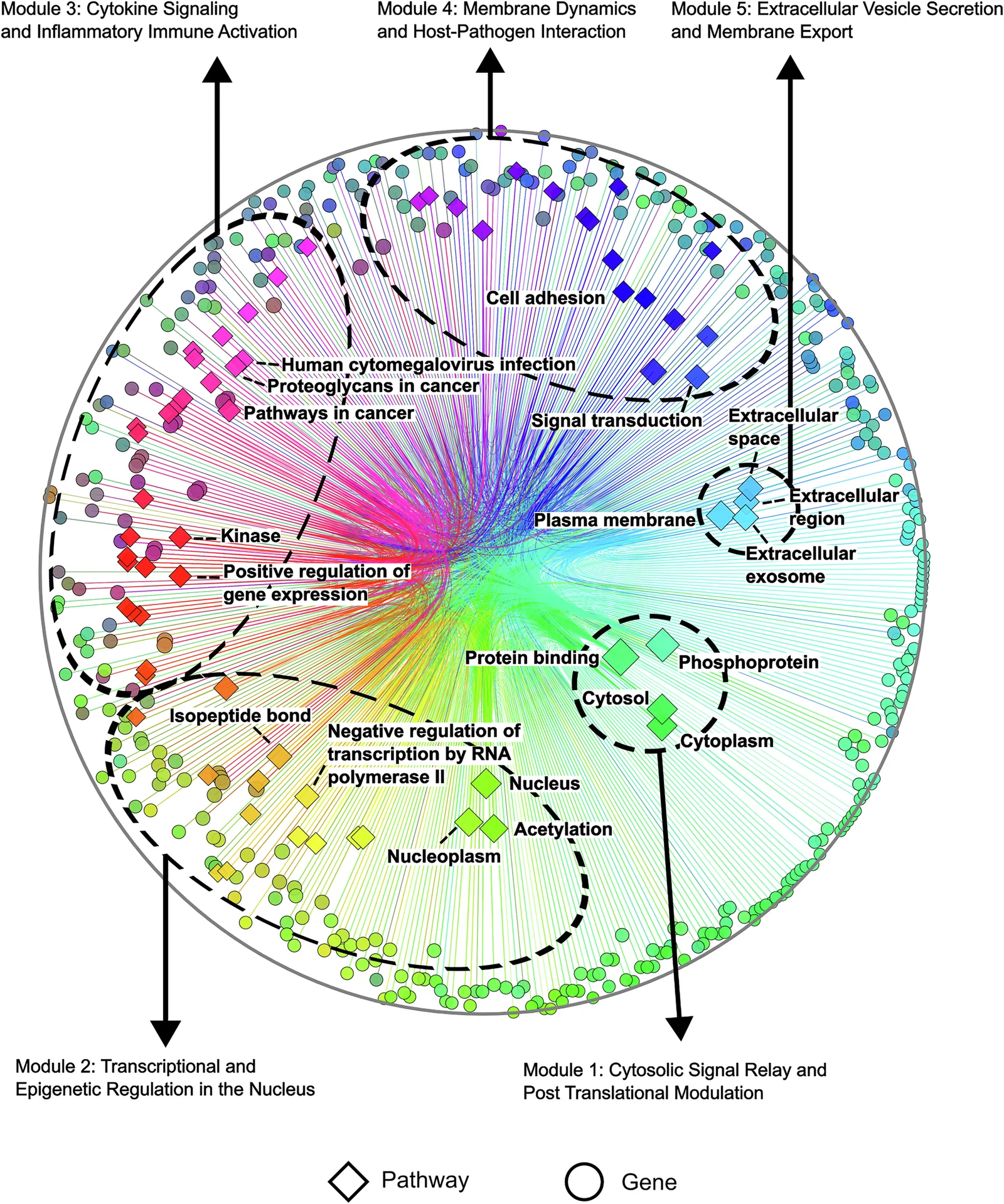
Hyperpathway visualization of genomic data. Hyperpathway visualization in the 2D disk of the gene-pathway interactions. Genes and enriched pathways are visualized in a hyperbolic space embedding. Nodes representing pathways are colored using a continuous angular color gradient, such that similar angular coordinates correspond to similar colors, while genes are assigned blended colors derived from the pathways to which they are connected. Pathway are scaled according to the number of enriched genes they contain, whereas genes are sized by their degree (number of pathway connections) in the bipartite network. Visually identified modules are marked by dashed ellipses, highlighting the set of pathways belonging to each module. Note that for clarity only the pathways are enclosed within the dashed ellipses; associated molecules may extend beyond the ellipses.

**Fig. 3:**
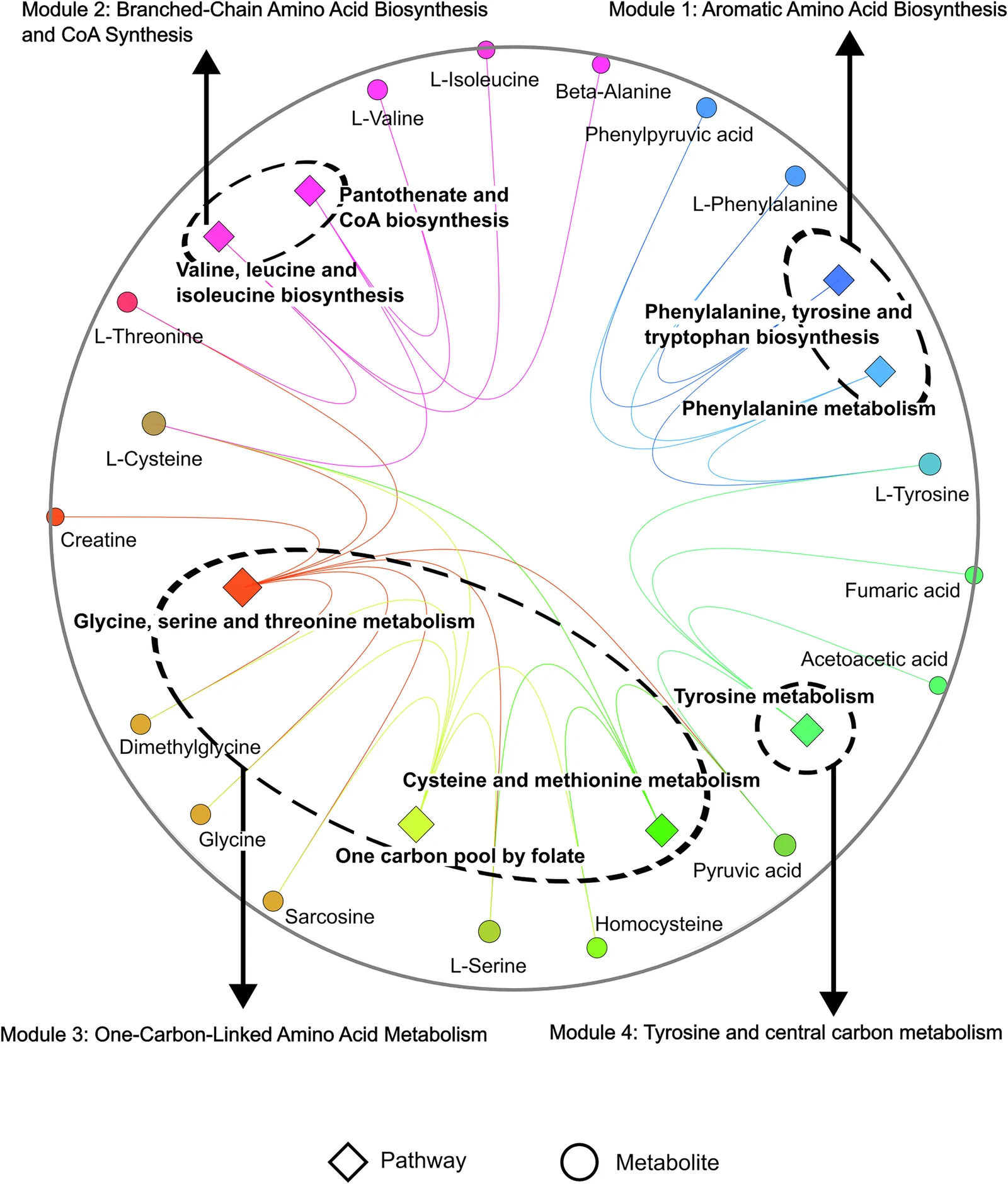
Hyperpathway visualization of metabolic data. Hyperpathway visualization in the 2D disk of the metabolite-pathway interactions. Metabolites and enriched pathways are visualized in a hyperbolic space embedding. Nodes representing pathways are colored using a continuous angular color gradient, such that similar angular coordinates correspond to similar colors, while metabolites are assigned blended colors derived from the pathways to which they are connected. Pathway are scaled according to the number of enriched metabolites they contain, whereas metabolites are sized by their degree (number of pathway connections) in the bipartite network. Visually identified modules are marked by dashed ellipses, highlighting the set of pathways belonging to each module. Note that for clarity only the pathways are enclosed within the dashed ellipses; associated molecules may extend beyond the ellipses.

**Fig. 4:**
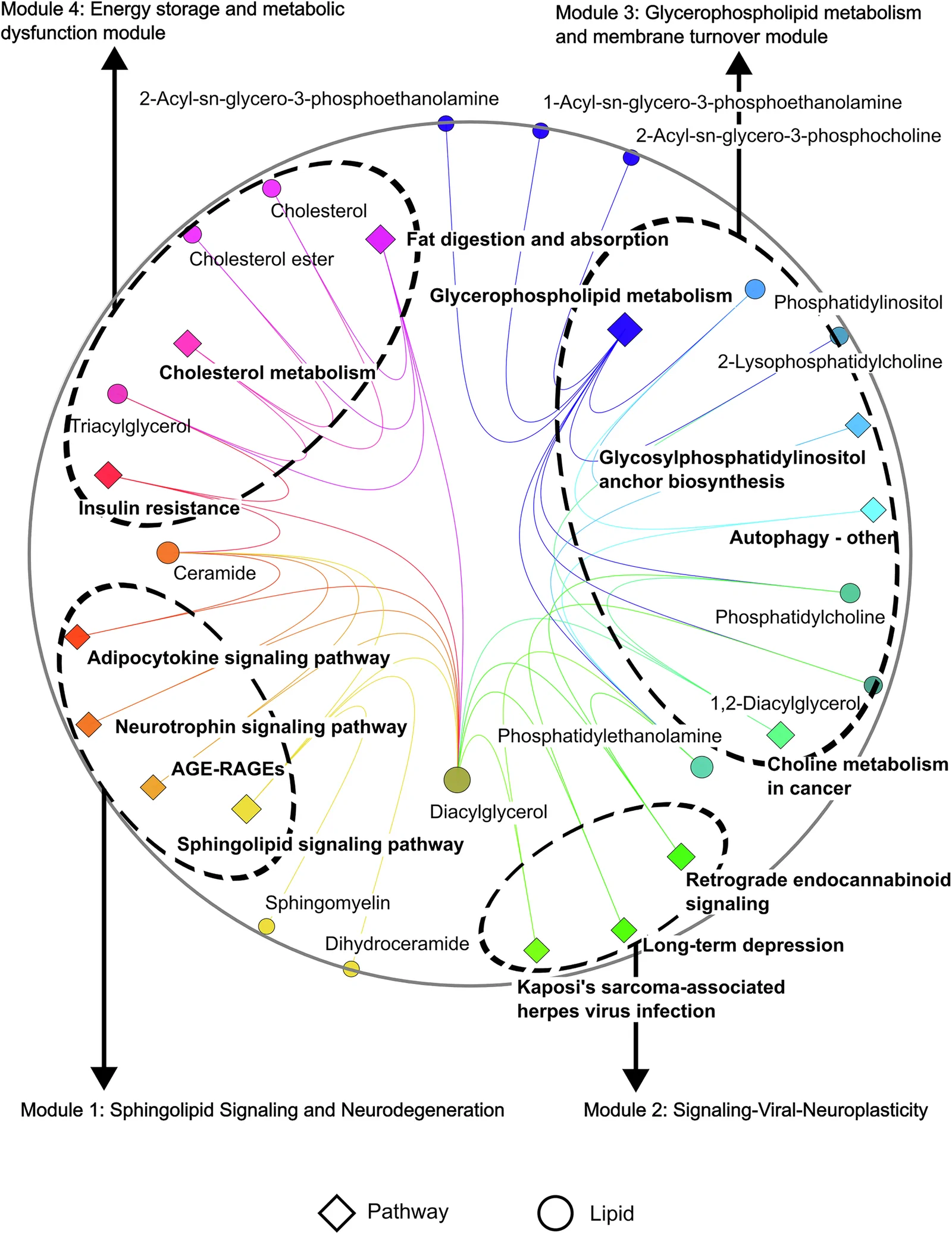
Hyperpathway visualization of lipidomic data. Hyperpathway visualization in the 2D disk of the lipid-pathway interactions. Lipids and enriched pathways are visualized in a hyperbolic space embedding. Nodes representing pathways are colored using a continuous angular color gradient, such that similar angular coordinates correspond to similar colors, while lipids are assigned blended colors derived from the pathways to which they are connected. Pathway are scaled according to the number of enriched lipids they contain, whereas lipids are sized by their degree (number of pathway connections) in the bipartite network. Visually identified modules are marked by dashed ellipses, highlighting the set of pathways belonging to each module. Note that for clarity only the pathways are enclosed within the dashed ellipses; associated molecules may extend beyond the ellipses.

Figure 1f shows the final step that visualizes the pathway–molecule interaction network in a 2D hyperbolic disk using a native hyperbolic representation^20^, with nodes colored according to the selected coloring mode (see Supplementary Fig. 19). In the 2D hyperbolic disk representation, radial coordinates are associated with node degree centrality. Nodes located towards the center of the disk have higher relevance in the network hierarchical organization, and are expected to play a pivotal role in the communication architecture of the system. In this study, they may indicate molecules of central hierarchical relevance across many enriched pathways, or pathways of higher relevance with respect to a certain subset of molecules. Angular coordinates are associated with node similarity: nodes that share a similar network neighborhood take very close angular coordinates in the geometric space, and have a similar role in the functioning of the system. These pathways typically share a similar subset of enriched molecules and collectively form a mesoscale structure, referred to as a ‘network community’ in social science and as ‘network module’ in systems biology^28^. The innovation of this Hyperpathway visualization is that it can display groups of pathways and molecules that co-locate in similar angular sectors of the 2D hyperbolic disk representation. This can reveal the presence of distinct network modules (see Figs. 2–4), each differentiated by their pathway–molecule components, which contribute in varied ways to the mechanisms driving the phenotype investigated in the omics study. Moreover, Hyperpathway can be used not only to represent the output of PEA, but also to represent any bipartite network (see Supplementary Fig. 18). The link for the open-access webtool to generate figures using Hyperpathway is: https://hyperpathways.org/. The command-line tool is also available at the GitHub page (see Code availability).

In the next sections, we provide real-world examples in omics data science that demonstrate the practical applicability of Hyperpathway in revealing and visualizing biological pathways, molecular entities (genes, lipids, and metabolites), and their interconnections. Across all examples, the layout consistently exhibits a clear bipartite architecture, where molecular entities (circle-shaped nodes) connect solely to pathway nodes (diamond-shaped nodes), accurately preserving the structural properties of the original output of the PEA analysis.

### Hyperpathway visualization of genomic data

For functional annotation, we used the *demolist2* gene set supplied by DAVID (Database for Annotation, Visualization, and Integrated Discovery)^5^, a widely used web-based tool (https://davidbioinformatics.nih.gov/) that provides comprehensive annotation and over-representation analysis across diverse biological categories. *demolist2* gene set (see Supplementary Data, genomic signature for Table 1) originates from the microarray study of Cicala et al^29^. profiling gp120-induced transcriptional responses in human peripheral blood mononuclear cells (PBMCs) and monocyte-derived macrophages (MDMs). This curated list of gp120-responsive human genes served as the input for our enrichment analysis. The resulting table of significantly enriched pathways was subsequently used as input for the Hyperpathway visualization (Supplementary Data, Table 1). At a significance threshold of *p*-value < 0.05, 38 pathways were enriched after Bonferroni correction, 68 after Benjamini-Hochberg correction, and 403 before multiple testing correction, i.e., uncorrected.

Figure 2 reports the Hyperpathway visualization of the significantly enriched gene-pathway interaction network, including only the 68 pathways retained after Benjamini-Hochberg correction, using the similarity coloring mode. We observe that dense network modules emerge around key biological processes. For example, one angular sector was densely populated with pathways involved in “Transcriptional and epigenetic regulation in the nucleus”, creating a module (module 2, Fig. 2) including *positive regulation of transcription by RNA polymerase II*, *DNA-binding transcription activator activity*, and *chromatin*. Another angular sector revealed a module (module 3, Fig. 2) of grouped pathways associated with “Cytokine signaling and inflammatory immune activation”, including *NF-κB*, *TNF*, *IL-17* signaling, *cytokine activity, malaria, and hepatitis B*. This inflammatory hub integrates multiple cytokine and pathogen-induced signaling pathways central to immune response, stress adaptation, and inflammation-associated disease. Another angular region revealed a module (module 4, Fig. 2) of “Membrane dynamics and host-pathogen interaction”, such as *Amoebiasis, Legionellosis*, *Cell adhesion, Calcium signaling*, and *glutamatergic synapse*, highlighting the spatial co-localization of genes involved in membrane-associated signaling, pathogen entry, and intercellular communication.

The similarity coloring mode further facilitates the identification of these densely connected regions, which emerge as network modules, thereby visually reinforcing that the Hyperpathway embedding captures functional proximity through angular organization and biological specificity along radial coordinates. Notably, genes located at higher radial coordinates tend to exhibit tighter pathway specificity, suggesting that radial stratification in the embedding encodes a biologically meaningful hierarchy. Supplementary Figure 2 reports the extended Hyperpathway visualization including all 403 pathways before multiple testing correction.

### Hyperpathway visualization of metabolomic data

For the metabolomics example, we used the demo dataset (see Supplementary Data, metabolomic signature Table 2) provided by MetaboAnalyst 6.0^30^ (https://www.metaboanalyst.ca/MetaboAnalyst/upload/PathUploadView.xhtml), which contains a mixed set of common metabolites, amino acids, and environmental chemicals. Running this list through the Pathway Analysis module yielded a set of significantly enriched metabolic pathways across multiple biochemical categories. The resulting output table (Supplementary Data, Table 2), listing pathways and their associated metabolites along with adjusted *p*-values, served as input for our subsequent Hyperpathway visualization. At a significance threshold of *p* < 0.05, 6 pathways were enriched after Bonferroni correction, 8 after Benjamini-Hochberg adjustment, and 16 before multiple testing correction.

Figure 3 reports the Hyperpathway visualization of the significantly enriched metabolite-pathway interaction network including only the 8 pathways retained after Benjamini-Hochberg correction, using the similarity coloring mode. Pathways are distributed along distinct angular branches, suggesting functional modularity. In the Hyperpathway representation, significant metabolic pathways often appear close to one another when they share common metabolites (precursors or intermediates). For instance, *Phenylalanine metabolism* and *Phenylalanine, tyrosine and tryptophan biosynthesis* are both located near *L-Phenylalanine*, *L-Tyrosine* and *Phenylpyruvic acid*, creating a network module (module 1, Fig. 3). Another spatial arrangement of interest is that formed by *Glycine, serine and threonine metabolism*, *Cysteine and methionine metabolism*, and *One carbon pool by folate* (module 3, Fig. 3). These pathways are enriched with a large number of common metabolites in their close neighborhood, resulting in their co-localization within the hyperbolic space. The co-localization of these nodes in the Hyperpathway representation highlights the presence of a network module, illustrating the central role of these pathways in one-carbon metabolism, methylation, and amino acid regulation, as well as their interconnectedness in maintaining cellular homeostasis^31^. Remarkably, the extended Hyperpathway visualization (Supplementary Information 1, Supplementary Fig. 3) by adding uncorrected significant pathways influences the overall network structure and spatial relationships between the pathways, rearranging the network modules in a broader context. Thus, even a new module related to cellular energy production (module 4, Supplementary Fig. 3 in Supplementary Information 1) can be identified.

The emergence of network modules in the geometric representation means that the layout reflects biochemical relationships: pathways that depend on the same core metabolites are placed nearby in the hyperbolic space. This feature supports the interpretability of the visualization, as it mirrors known biochemical connectivity in a geometric form.

### Hyperpathway visualization of lipidomic data

We used lipidomic data from a multi-omics study^32^ involving 2,608 individuals across three type 2 diabetes (T2D) cohorts. The dataset includes high-quality measurements of 162 lipid species, including triacylglycerols and sphingomyelins, after rigorous quality control and batch correction. The dataset of potential lipid markers for T2D (see Supplementary Data, lipidomic signature for Table 3) was subsequently analyzed using LIPEA (Lipid Pathway Enrichment Analysis)^14^, a web-based tool (https://hyperlipea.org/home). LIPEA performs over-representation analysis to identify biological pathways enriched in lipid signatures. The analysis was conducted using the default background for *Homo sapiens*, meaning all lipids annotated in human metabolic pathways were considered as the reference set from which the input signature was derived. LIPEA returns a list of enriched pathways sorted by adjusted *p*-values (Bonferroni, Benjamini-Hochberg). At a significance threshold of *p*-value < 0.05, 4 pathways were enriched after Bonferroni correction, 14 after Benjamini-Hochberg adjustment, and 38 before multiple testing correction (Supplementary Data, Table 3).

Figure 4 reports the Hyperpathway visualization of the significantly enriched lipid-pathway interaction network including only the 14 pathways retained after Benjamini-Hochberg correction, using the similarity coloring mode. Central lipids such as Diacylglycerol (DAG) and Phosphatidylethanolamine (PE) emerge as key hubs, linking multiple network modules involved in various critical biological processes including membrane dynamics, energy storage, and inflammation. Pathways like *Glycerophospholipid metabolism* and *Sphingolipid signaling pathway* appear closer to the core, suggesting higher connectivity or shared lipids with multiple other pathways. Glycerophospholipids are crucial for maintaining membrane integrity, mediating insulin signaling, and regulating inflammatory responses; all of which are dysregulated in type 2 diabetes (T2D)^33,34^.

G*lycerophospholipid metabolism* and *Choline metabolism in cancer* co-locate together, forming a network module (module 3, Fig. 4), which shares several lipid species. This is biologically coherent, as choline-containing phospholipids play roles in both pathways. Choline-containing phospholipid metabolisms were shown to be altered in incident T2D groups^33–35^

Also, key metabolic pathways relevant to T2D, like *Insulin resistance*, *Cholesterol metabolism*, and *Fat digestion and absorption*, are not only significantly enriched but also densely connected (module 4, Fig. 4) to multiple central lipids (e.g., Triacylglycerol, Cholesterol), reinforcing their relevance to the lipidomic signature of T2D progression. Their positioning near the core of the network reflects robust and multi-lipid associations.

In contrast, some pathways, although statistically significant, are connected to the lipid signature via a single lipid node. These isolated connections, while valid by enrichment statistics, may reflect more tenuous or specific biochemical relationships and thus benefit from cautious interpretation. The presence in peripheral or weakly connected regions (Supplementary Information 1, Supplementary Figs. 2–4) of numerous significant uncorrected pathways provides a background for comparison and highlights the structural coherence of the top-enriched terms. The Hyperpathway representation for lipidomic data offers a visual summary of how lipidomic signals map onto functionally distinct yet disease-relevant biochemical axes in T2D. Supplementary Figure 4 reports the extended Hyperpathway visualization including all 38 pathways before multiple testing correction (i.e., uncorrected).

### Comparison with existing network visualization approaches

To assess the added value of hyperbolic geometry, we compared the Hyperpathway embedding with commonly used network visualization approaches implemented in Cytoscape^36^, including force-directed (edge betweenness), organic, and radial layouts, applied to identical pathway–molecule bipartite networks. Force-directed and organic layouts effectively preserve local connectivity but lack a global organizing principle: node positions are driven primarily by local edge forces, leading to spatial overlap between biologically distinct pathways, dominance of highly connected molecules, and rapid loss of interpretability as network size increases (Supplementary Information 1, Supplementary Figs. 5–7 and 11–13). Radial layouts provide a clear global frame but impose an arbitrary geometry in which pathway relevance, connectivity, and enrichment are not reflected in node position, resulting in extensive edge congestion and limited modular resolution (Supplementary Information 1, Supplementary Figs. 8–10). In contrast, the Hyperpathway embedding integrates network topology and pathway relevance directly into geometry. Highly connected or strongly enriched pathways are positioned closer to the center, whereas weakly connected or marginal pathways are consistently displaced toward the periphery. Functionally related pathways occupy coherent angular sectors, enabling clear visual separation of biological modules while preserving global context. This hierarchical and modular organization reduces visual clutter, improves scalability, and facilitates rapid prioritization of biologically central pathways, representing a key advantage over conventional force-directed, organic, and radial layouts for exploratory analysis of complex pathway–molecule networks.

### Web-based versus local performance, and computational speedup of leaf removal and reinsertion

#### Computational speedup of the leaf removal and reinsertion pipeline (Fig. 5a)

We first benchmarked the computational speedup achieved by the leaf removal and post-hoc reinsertion pipeline (Innovation 3a–3b) against direct full-network embedding, across a progressive range of leaf node proportions (0–100% of leaves reintroduced), on two machines: a laptop (16 GB RAM) and a workstation (256 GB RAM). At 0% leaves, the original algorithm already operates on the full original network (inclusive of all leaf nodes) requiring approximately 3 min 15 s on the laptop and 1 min 48 s on the workstation. The leaf removal and reinsertion pipeline at this same point embeds only the pruned core network, with no leaves yet reinserted, achieving a marginally lower computation time of approximately 3 min 13 s on the laptop and 1 min 46 s on the workstation. This is a negligible difference reflecting the fact that leaves represent only ~2% of the total network size at this baseline. The speedup becomes increasingly pronounced as the proportion of leaves progressively added to the original algorithm’s input grows from 0 to 100%, inflating its network size and embedding cost monotonically, while the accelerated pipeline’s computation time remains near-flat since post-hoc reinsertion runs in *O*(|${V}_{L}$ | ), where ${V}_{L}$ is the number of leaves (see Supplementary Information 1—Algorithm 5) and adds negligible overhead regardless of the number of reinserted leaves. At full leaf reinsertion (100%), the original algorithm reaches 10 min 39 s on the laptop and 5 min 28 s on the workstation, while the pipeline remains at approximately 3 min 20 s and 1 min 50 s respectively, yielding a speedup of **×**3.26 on the laptop and **×**3.03 on the workstation. Critically, this speedup is achieved without any loss of geometric fidelity: the hyperbolic coordinates of all core-network nodes remain unchanged, and the reinserted leaves are placed deterministically using local angular rules anchored to their topological parent (see “Methods”—Innovation 3b). These results confirm that the leaf removal and reinsertion strategy provides consistent, hardware-independent acceleration, making Hyperpathway tractable for large-scale pathway–molecule networks.

**Fig. 5:**
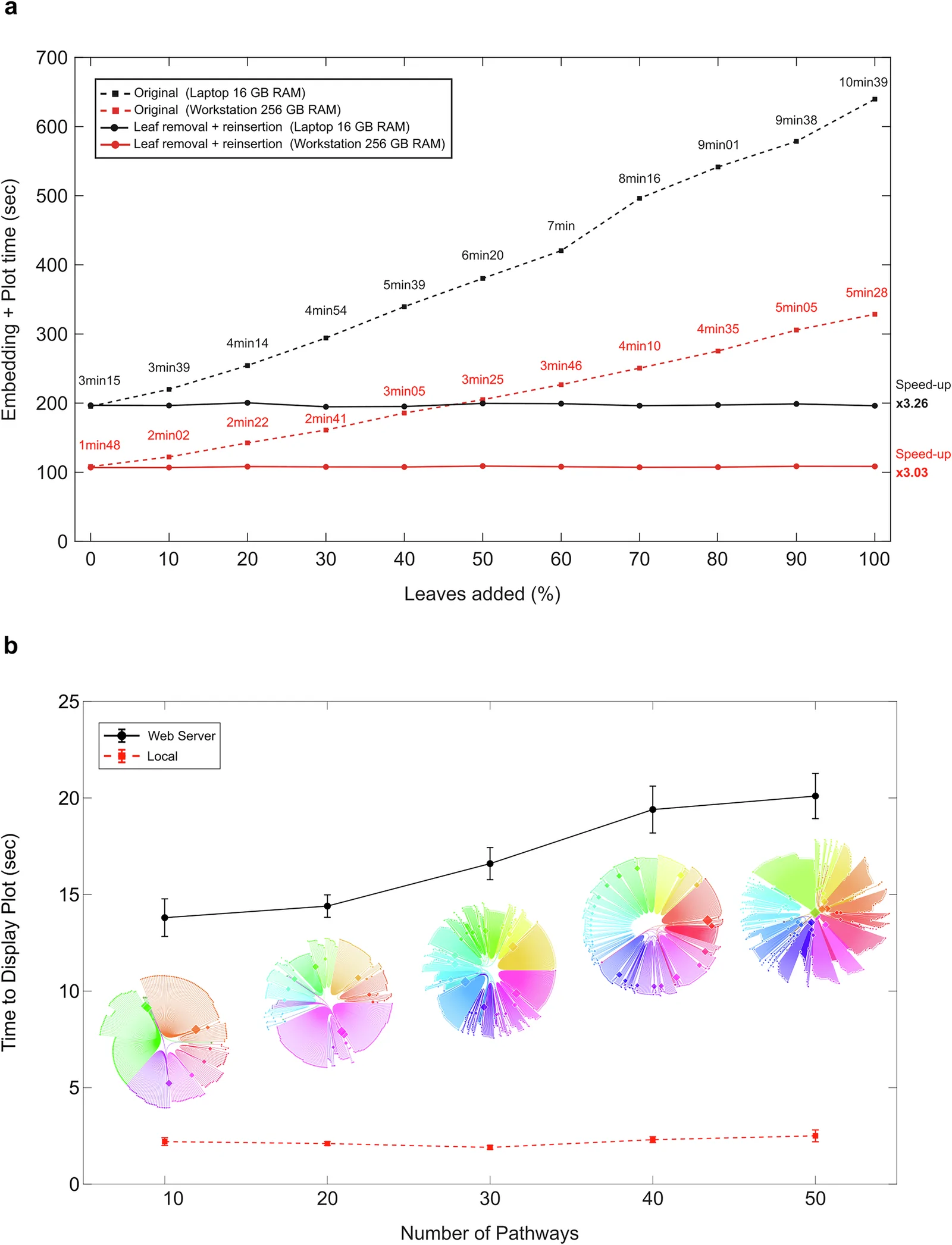
Computational performance of the Hyperpathway framework. **a** Total computation time (embedding + plot rendering) as a function of the proportion of artificial leaf nodes progressively reintroduced into the network (0–100%), comparing the original Hyperpathway algorithm against the proposed leaf removal and post-hoc reinsertion pipeline, benchmarked on two machines: a laptop (16 GB RAM, black) and a workstation (256 GB RAM, red). Dashed lines denote the original algorithm; solid lines denote the leaf removal + reinsertion pipeline. Annotated time labels indicate absolute runtimes at each measurement point. At 100% leaves, the leaf removal and reinsertion strategy achieves a speedup of ×3.26 on the laptop and ×3.03 on the workstation relative to direct full-network embedding. **b** Time to display pathway network plots as a function of the number of Reactome pathways (10–50), comparing local execution on the workstation with the Hyperpathway web application hosted on Azure Web Server (see Methods). Points show mean display time across 10 runs, with error bars indicating standard error. Black solid lines denote web-based performance, and red dashed lines denote local performance. Representative visualizations for each pathway size are shown.

#### Embedding + display time comparison between web and local execution at practical pathway scales (Fig. 5b)

We then benchmarked performance under realistic use cases in which users are expected to explore a limited number of pathways simultaneously (10–50 pathways). At these scales, the web application managed interactive visualization within 20 seconds, with moderate increases in rendering time, while local execution remained consistently fast. To evaluate performance at larger scales, we extended the benchmarking to include subsamples of 100, 500, 1000, 1500, 2000, 2500, and the complete Reactome Pathways Gene Set with 2810 pathways (Supplementary Information 1, Supplementary Fig. 14). As pathway number increased, the time required to render pathway networks on the web server rose, reaching nearly 5 minutes for the full dataset. In contrast, local execution on the workstation exhibited a substantially lower and more gradual increase in rendering time across all tested scales. We want to emphasize that visualizations involving more than 50 pathways are not frequent in practice, as interpreting relationships between pathways and genes becomes increasingly challenging at this scale. Consequently, we expect that most users will benefit from the web tool without the need to run Hyperpathway locally on their desktop (see Code availability).

## Discussion

Hyperpathway is designed to deliver a fast and informative visual summary of bipartite pathway–molecule relationships derived from omics data through Pathway Enrichment Analysis (PEA). The method takes as input a tabular PEA output in which the first two columns specify pathways and their associated molecules, and may optionally include enrichment statistics (uncorrected or multiple-testing–adjusted p-values) and/or user-defined categorical annotations. It projects the pathway–molecule interaction network onto a two-dimensional hyperbolic disk and applies flexible coloring schemes to pathway nodes based on network geometry (similarity- and hierarchy-based encodings), statistical significance following multiple-testing correction, or user-defined categorical annotations. The radial coordinate in the 2D Hyperpathway representation is associated with the hierarchical role of a node; hence, central nodes have higher hierarchical relevance. The angular coordinate instead reflects the level of similarity between the nodes; thus, nodes in the same angular sector are more closely associated. Hyperpathway generates a mapping that transforms the molecule-pathway interaction network’s topological hierarchy and similarity into geometric distances, thereby revealing and deepening our understanding of its complex modular structure. Functional modules emerge as ensembles of nodes co-localized within similar angular coordinate sectors, while integration across distinct modules is mediated by nodes exhibiting higher radial centrality.

The user can choose between two options: (i) rendering up to two hyperbolic embeddings of the same pathway–molecule network on a single page, each displayed with a distinct coloring scheme if needed, from which subnetworks can be interactively extracted; or (ii) rendering up to two hyperbolic embeddings of a preconstructed bipartite network (see Supplementary Fig. 18), again with different coloring schemes and identical subnetwork extraction capabilities.

This dual-view output provides a multi-layered perspective on the data and is particularly advantageous for the exploration and interpretation of multi-omics networks, or complex systems in general. The use of hyperbolic geometry ensures that both global context and local details are preserved, enabling an intuitive interpretation of hierarchical and modular structures in biological networks. Based on the observed performance characteristics, we recommend local execution for exploratory analysis of very large pathway collections or general bipartite networks already prepared for visualization.

In future development, we plan to extend Hyperpathway with additional post-visualization algorithms, such as automated node clustering and community detection, to provide users with computational support for identifying putative network modules. For the initial release, however, our focus remains on simplicity and user-friendliness through data visualization, with subsequent expansions guided by user feedback. In terms of limitations, we could argue that incorporating direct molecule–molecule interactions from curated databases into the PEA bipartite network is feasible. However, this would complicate and destabilize the network’s structure, disrupting its bipartite organization and deviating from the outcomes of the original PEA analysis. Introducing such links would shift the visualization’s focus away from a direct interpretation of the PEA output, resulting in a more intricate and less tractable landscape. This presents a distinct representational challenge that warrants investigation in future studies. Currently, Hyperpathway operates exclusively at the molecular scale, focusing on relationships between pathways and entities such as genes, proteins, lipids, metabolites, etc. This focus is already valuable for understanding system-level organization and deriving mechanistic hypotheses on pathway-molecule interactions underlying molecular signatures. However, we recognize a critical need to extend the scope of Hyperpathway beyond molecule-level data to encompass sample-level embeddings. This expansion would allow pathway-based representations of individual samples, creating the potential for personalized pathway fingerprints. In clinical and translational settings, transforming thousands of raw molecular measurements into a smaller, interpretable set of pathway activities per sample is a desirable goal. This reduction can enhance interpretability, support clustering or stratification of patients, and ultimately aid in personalized therapeutic decisions. Sample-scale analysis would also enable the study of dynamic changes in pathway activity over time or in response to treatment, making Hyperpathway a more versatile tool for network medicine. We envision integrating this sample-level functionality by projecting sample profiles onto the existing pathway hyperbolic space using similarity measures, such as pathway activity scores. This would allow Hyperpathway to serve as a unified interface for both molecule-to-pathway and sample-to-pathway interpretations, maintaining its visual and topological strengths while broadening its analytical capabilities. In summary, Hyperpathway introduces an innovative way to visualize and interpret functional pathway enrichment results through the lens of hyperbolic geometry. While its current utility lies in molecular-scale visualization, future extensions to sample-level embedding could position it as a powerful tool for personalized omics analysis and pathway-informed decision-making in precision medicine.

## Methods

### Hyperpathway architecture

Hyperpathway is a web-based application compatible with all major browsers, hosted on Microsoft Azure Web Services. The backend computational engine is implemented in Python 3.11, leveraging scientific computing packages including NumPy, SciPy, Pandas, and Plotly for network analysis and visualization, and was designed to accommodate up to 10 concurrent users. The web interface is built using Streamlit, providing an interactive user experience.

The computational pipeline processes pathway enrichment analysis (PEA) results through six sequential steps (see Fig. 1a): (i) conversion of tabular PEA outputs into bipartite network representations, (ii) artificial linking strategy if disconnected components in a graph, (iii) leaf removal (iv) coalescent embedding into hyperbolic space, (v) post-hoc leaf reinsertion, and (vi) interactive visualization in the 2D hyperbolic disk with customizable coloring schemes and export capabilities.

Users can upload PEA results in standard formats (Excel or CSV) containing pathway names, enriched molecules, and statistical significance values (optional). Alternatively, users can directly provide pre-constructed bipartite adjacency lists with optional custom node and edge coloring specifications.

All visualizations are generated in real-time and can be explored interactively through pan, zoom, and node selection features. Users can export high-resolution figures (SVG, PDF and PNG) for publication. Additionally, the application provides downloadable Excel files containing node coordinates, network topology (edge lists), and node attributes, enabling downstream analysis in external tools such as Cytoscape or custom scripting environments. Visualization sessions persist during active use but do not require user accounts or long-term data storage on the server.

For researchers requiring batch processing or integration into computational pipelines, the complete Hyperpathway source code is openly available on GitHub https://github.com/biomedical-cybernetics/Hyperpathway, enabling local installation and customization. The web interface can be accessed at https://hyperpathways.org/.

### Genomic data

Gene-level functional enrichment analysis was performed using DAVID (Database for Annotation, Visualization, and Integrated Discovery)^5^; https://davidbioinformatics.nih.gov/summary.jsp. All analyses were run with default parameters, which include the modified Fisher’s exact test (EASE score = 0.1), the human genome as background, and both Benjamini-Hochberg and Bonferroni corrections for multiple testing. As input, we used DAVID’s built-in example gene list *“demolist2”*, which consists of 403 genes up-regulated in peripheral blood mononuclear cells (PBMCs) from four healthy donors exposed to HIV gp120 envelope proteins^29^. In that study, Cicala et al. profiled transcriptional and protein responses of PBMCs and monocyte-derived macrophages (MDMs) stimulated with purified HIV gp120. Differentially expressed genes were identified using Affymetrix microarrays and Significant Analysis of Microarrays (SAM), producing a robust gene list that has since been widely used for enrichment studies. This curated gp120-responsive gene signature constitutes the *demolist2* dataset used as input for our DAVID enrichment analysis. The resulting enrichment table (Supplementary Data, Table 1) was subsequently visualized with Hyperpathway.

### Metabolomic data

We used MetaboAnalyst 6.0^30^, a user-friendly web-based tool for statistical and functional interpretation of metabolomics data. The tool supports a wide variety of univariate or multivariate statistical and machine learning methods to identify important features and patterns. It supports Pathway Analysis for 26 organisms as well as enrichment analysis of ~9000 metabolite sets.

The example data in MetaboAnalyst 6.0 corresponds to a mixed list of common metabolites and amino acids (e.g., glycine, serine, valine, etc), and environmental chemicals (e.g., arsenic, cadmium, lead, benzene, etc). This mixed set is not tied to a single published study, but rather serves as a demo dataset to showcase how its pathway analysis can handle heterogeneous molecular inputs.

We ran the pathway analysis module with the example data provided by the webtool to identify biological pathways significantly enriched with metabolites of interest. The resulting table (Supplementary Data, Table 2) of enriched pathways served as input for our subsequent hyperpathway visualization.

### Lipidomic data

Lipidomic data were obtained in the study^32^ from plasma samples of 2,608 individuals across three type 2 diabetes (T2D) cohorts. Lipid profiling was performed using the Lipotype Shotgun Lipidomics platform on a QExactive mass spectrometer (Thermo Scientific) following standardized guidelines. A total of 614 lipid species were initially measured; after quality control and batch correction using reference plasma samples, 162 high-quality lipid species were retained for analysis. Lipid identification and quantification were conducted using LipidXplorer-based software, with stringent criteria on signal-to-noise ratio and blank comparisons. The resulting lipidomic data were analyzed on the web-based tool called LIPEA (Lipid Pathway Enrichment Analysis)^14^, a new valid resource for biologists and physicians to mine pathways significantly associated with a set of lipids, helping them to discover whether common and collective mechanisms are hidden behind those lipids. LIPEA performs over-representation analysis to identify biological pathways enriched in lipid signatures. The analysis was conducted using the default background for *Homo sapiens*, meaning all lipids annotated in human metabolic pathways were considered as the reference set from which the input signature was derived. LIPEA returns a list of enriched pathways. The resulting table (Supplementary Data, Table 3) of enriched pathways served as input for our subsequent hyperpathway visualization.

### Hyperpathway

The computational pipeline processes PEA results through six sequential steps (see Fig. 1a): (i) PEA-to-bipartite network conversion (Innovation 1); (ii) artificial linking strategy for disconnected components (Innovation 2); (iii) leaf removal (Innovation 3a); (iv) coalescent embedding into hyperbolic space; (v) post-hoc leaf reinsertion (Innovation 3b); and (vi) interactive visualization in the 2D hyperbolic disk. Each step is described in a dedicated subsection below

#### Innovation 1 - Conversion of the pathway analysis outputs into bipartite network representation

Based on the output of the PEA, the first entry is the list of significant pathways and the second entry is the subset of enriched molecules for each pathway. We created an algorithm to convert these two entries into a PEA bipartite network. The time complexity of the algorithm is, in most cases *O((P* + *M)*^2^*)*, with P as the number of pathways and M as the number of unique molecules. The pseudo-code is provided in Supplementary Information 1, Algorithm 1. The bipartite network presents pathway and molecule (e.g., metabolites, lipids, genes, etc.) nodes and it links molecular entities to the biological pathways in which they are significantly enriched. This bipartite structure is then used as input for coalescent embedding.

#### Innovation 2—Artificial linking strategy between separated components

A key methodological innovation of Hyperpathway is its ability to embed pathway-molecule association networks that consist of multiple separated components, a scenario frequently encountered in real-world biological datasets due to sparsity. Standard embedding algorithms often fail or provide misleading layouts when applied to disconnected graphs. To address this, we developed a procedure that automatically links graph components selectively before embedding, thereby allowing coherent hyperbolic layout without distorting the intrinsic topology. Given an input matrix encoding pathway-molecule associations, we first computed the adjacency graph and identified the number of graph components. If the graph consists of a single connected component, we proceed directly with coalescent embedding as described above. If multiple components are detected, we apply an artificial linking strategy to connect components to each other with negligible bias to the network structure. For each component, we identify two representative nodes: one for the pathway class (pathway anchor) and one for the molecule class (molecule anchor). Each of these two nodes is selected as the one with the highest degree with respect to its own class within the component. If multiple nodes have the same highest degree inside a class, we only select the first one encountered in the respective class. Each component is then connected to the rest of the network by linking its pathway anchor to the molecule anchor with the highest degree across the other components. This procedure ensures that only a minimal number of inter-component links are introduced, resulting in a bipartite graph augmented with exactly *c* − *1* links, where *c* is the number of original disconnected components. This minimally augmented graph is then processed by the coalescent embedding algorithm, allowing for a coherent global layout that maintains the distinct visual identity of each original module. These artificially introduced links are not rendered in the final visualization; they serve exclusively as structural bridges for embedding, leaving the intrinsic bipartite topology intact. The time complexity of the artificial linking strategy described is *O(N*^*2*^ + *C⋅ N* + *C*^*2*^*)*, where *C* is the number of connected components in the input graph, *N* and *E* denote the total number of nodes and edges, respectively. The details of the algorithm and its time complexity are in Supplementary Information 1, Algorithm 2.

#### Innovation 3a—Leaf removal prior to embedding

Coalescent embedding scales close to quadratically for sparse graphs, which is computationally sustainable also for embedding of large pathway-molecule bipartite networks. However, we propose a solution to improve running time that can help especially when implemented as an open webtool on servers. We introduce a leaf removal procedure applied prior to embedding. A leaf node is defined as any node of degree one. Such nodes are geometrically invariant: because their sole connection provides no angular constraint relative to any other node, they do not influence the latent geometry of the network and their removal does not alter the hyperbolic coordinates of any remaining node. Formally, let $G=(V,{E})$ be the connected bipartite graph produced after artificial linking. The leaf removal procedure identifies all degree-one nodes (which form the set ${V}_{L}$) in a single pass and removes them together with their incident edges (which form the set ${E}_{L}$), yielding a reduced core graph ${G}^{{\prime} }=({V}^{{\prime} } < \,V,\,{E}^{{\prime} } < {E})$. The core graph ${G}^{{\prime} }$ is then passed to the coalescent embedding algorithm, substantially reducing both memory requirements and computation time while preserving the topological structure that determines the hyperbolic geometry of the network. Overall, the time complexity is *O(V* + *E)*. The details of the algorithm and its time complexity are in Supplementary Information 1, Algorithm 3.

#### Coalescent embedding of the PEA bipartite network

Coalescent embedding^18^ is a class of topological-based machine learning algorithms for nonlinear unsupervised dimensionality reduction and embedding of networks in a geometric space, such as hyperbolic one. The approach always involves 4 steps: (1) pre-weighting links with topological rules that approximate the underlying network geometry; (2) computing non-linear dimension reduction; (3) calculating angular coordinates; and (4) calculating radial coordinates. In this study^18^, we implemented coalescent embedding considering the settings used in the original study, which offered excellent results in across many tests: Repulsion-attraction (RA1) pre-weighting (1), noncentered Isomap^37^ (ncISO) (2); Equidistant adjustment (EA) (3); log-based popularity fading formula (4). In this way, we can effectively embed the original network for visualization into 2D hyperbolic space, reconstructing the network organization according to its latent manifold structure. The pseudo-code is provided in Supplementary Information 1, Algorithm 4.

#### Innovation 3b–Post-hoc leaf reinsertion

Following coalescent embedding of the core graph ${G}^{{\prime} }$, all pruned leaf nodes in ${V}_{L}$ with their incident edges in ${E}_{L}$ are reinserted into the finalized layout in two steps: radial coordinate assignment and angular coordinate assignment. The pseudo-code is provided in Supplementary Information 1, Algorithm 5.

##### Radial coordinates

Radial coordinates are assigned globally to all nodes, including leaves, by fitting a power-law distribution to the degree sequence of the full original network G and applying the standard coalescent embedding radial rank formula (see Supplementary Information 1, Algorithm 4). Since leaf nodes have degree one, they receive the lowest-rank radial coordinates and are naturally placed toward the periphery of the hyperbolic disk, consistent with the radial-centrality relationship in hyperbolic space.

##### Angular coordinates

For each leaf $v\in {V}_{L}$ with unique topological neighbor (parent) $u\in {V\; \backslash }{V}_{L}$, the angular placement is anchored to u. First, the nearest neighbor w of the parent node u is identified among all core-network embedded nodes by minimizing the shorter circular arc in the RAA angular space:

$$w=\mathop{{argmin}}\limits_{j\in V\backslash {V}_{L},j\ne u}\min \left(\left|{\theta }_{j}^{{RAA}}-{\theta }_{u}^{{RAA}}\right|,\,2\pi -\left|{\theta }_{j}^{{RAA}}-{\theta }_{u}^{{RAA}}\right|\right)$$

The placement direction δ of w relative to u is then determined from their EA embedding angles ${\theta }_{w}^{{EA}}$ and ${\theta }_{u}^{{EA}}$ using the signed forward arc:

$$d=\left({\theta }_{w}^{{EA}}-{\theta }_{u}^{{EA}}\right)\mathrm{mod}2\pi$$

If d<π, node ${w}$ is counter-clockwise from ${u}\left(\delta =-1\right)$ and ${\theta }_{{center}}$ is placed at the midpoint of the clockwise arc away from w:

$${\theta }_{{center}}=\left({\theta }_{u}^{{EA}}+\frac{d}{2}\right)\mathrm{mod}2\pi \,{\rm{if}}d < \pi$$

If $d\ge \pi$, node ${w}$ is clockwise from ${u}\left(\delta =1\right)$ and ${\theta }_{{center}}$ is placed at the midpoint of the counter-clockwise arc away from w:

$${\theta }_{{center}}=\left({\theta }_{u}^{{EA}}-\frac{2\pi -d}{2}\right)\mathrm{mod}2\pi \,{\rm{if}}d\ge \pi$$

In both cases, ${\theta }_{{center}}$ is the midpoint of the arc directed away from the nearest neighbor, ensuring that the leaf fan occupies the least crowded angular sector around the parent. When a parent node has ${n}_{l}$ leaf children, they are distributed symmetrically as a fan centered at ${\theta }_{{center}}$:

$${\theta }_{v}^{(k)}={\theta }_{{center}}+\left(k-\frac{{n}_{l}-1}{2}\right)\cdot \frac{2\pi }{{N}_{{core}}},k=0,\cdots ,\,{n}_{l}-1$$

where ${N}_{{core}}=\left|{V\; \backslash }{V}_{L}\right|$ is the number of nodes in the core network $G{\prime}$ and $\frac{2\pi }{{N}_{{core}}}$ is the equidistant angular step of the EA embedding. The fan half-span is capped at π to prevent angular wrapping on very large leaf clusters. A small angular nudge of $0.1\times \,\frac{2\pi }{{N}_{{core}}}$ is applied to any leaf whose assigned angle coincides with an existing grid slot, and a final duplicate resolution pass ensures all angular coordinates across the full network are unique. This procedure runs in $O(\left|{V}_{L}\right|)$ time and leaves the coordinates of all embedded core nodes in $G{\prime}$ entirely unchanged, fully preserving the hyperbolic geometry of the core network while restoring a complete and topologically faithful representation of the original network G. Overall, the final complexity is *O*(${N}_{{core}}^{2}$ + *N log N)*.

#### Visualization of the embedded network in the 2D native hyperbolic disk

The time complexity of the visualization algorithm is *O(N* + *E)*, where *N* and *E* denote the total number of nodes and edges, respectively. The pseudo-code is provided in Supplementary Information 1, Algorithm 6.

##### Node representation and sizing

To preserve the bipartite structure, pathway nodes are displayed as diamonds, while molecular entities (genes, metabolites, lipids, etc) are displayed as circles. Node sizes are scaled according to degree centrality using a non-linear power transformation (${{degree}}^{0.6}$), enhancing separation between highly connected nodes and peripheral nodes while limiting excessive size inflation.

##### Coloring schemes

Hyperpathway implements five complementary coloring modes that highlight distinct aspects of pathway–molecule organization.

(1) Similarity coloring (angular coordinate-based).

Pathway nodes are assigned colors using a continuous hue–saturation–value (HSV) gradient mapped to their angular coordinates ($\theta \,\epsilon \,\left[0,\,2\pi \right]$) such that nodes with similar angular positions receive similar hues. Molecule nodes inherit blended colors computed as the RGB average of their connected pathway neighbors.

(2) Hierarchy coloring (radial coordinate-based).

This scheme applies a blue-to-red gradient based on node degree centrality, with low-degree nodes colored blue and highly connected nodes colored red. Intermediate colors are computed via piecewise linear interpolation in RGB space across four quantiles of the degree distribution.

(3) Label-based coloring (category-based).

Nodes may be colored according to categorical annotations provided in the pathway enrichment input table (e.g., pathway class, functional community, tissue specificity, or disease association). Pathway nodes sharing the same label receive identical colors selected from a maximally distinct HSV palette generated using the golden ratio ($\varphi \approx 0.618$). Molecule nodes are colored as the average of their connected pathway colors, while unlabeled nodes are displayed in gray.

(4) Pathway significance coloring.

Pathway nodes are colored according to statistical significance after multiple testing correction: red indicates Bonferroni-corrected significance, orange indicates Benjamini-Hochberg correction, gray indicates uncorrected significance (*p* < 0.05). Molecule nodes are uniformly colored green to preserve visual separation between node types.

(5) Preference coloring (user-defined).

This mode enables user-defined customization via uploaded color files specifying exact colors for individual nodes and/or edges. Colors may be provided in HEX (#FF0000), space-separated RGB (255 0 0), or normalized RGB (1 0 0) formats. Nodes and edges without user-defined colors default to gray.

#### Edge rendering and coloring

Edges are rendered as geodesic arcs in the Poincaré disk model. To reduce visual clutter in dense networks, edge rendering is capped at 20 000 randomly sampled edges when this threshold is exceeded.

Edge coloring depends on the selected node coloring scheme. In Pathway Significance mode, edges are uniformly gray. In gradient-based modes (Similarity, Hierarchy, and Label-based coloring), edges inherit the color of their connected pathway node. In Preference mode, user-specified edge colors are applied when provided; otherwise, edges default to gray. Edge opacity is user-adjustable (default 0.6).

### User interface

Supplementary Figures 15–17 describe the Hyperpathway user interface step-by-step using the above-mentioned lipidomic data. Screenshots from the webtool specific to uploading, selecting are annotated with a red spot, whereas those specific to visualization are annotated with a green spot.

### Reactome pathways gene set used for benchmarking

Pathway data were derived from the Reactome Pathways Gene Set (https://reactome.org/download-data—downloaded in November 2025) which compiles all curated Reactome pathways (for a given species, e.g., human) and all genes/proteins annotated to those pathways. To assess visualization performance across increasing dataset sizes, random subsamples of pathways were generated at predefined scales. For the primary benchmark shown in Fig. 5b, subsamples contained 10, 20, 30, 40, or 50 pathways. Additional benchmarks were generated for larger scales (100, 500, 1500, 2000, 2500, and the complete pathway set), for which an equivalent figure without representative visualizations is reported. For each pathway size, independent random sampling was repeated 10 times to minimize sampling bias, and each subsample was saved as a separate Excel file.

#### Benchmarking procedure

Each subsampled Excel file was processed identically under two execution environments: (i) a local workstation and (ii) the deployed web application hosted on Microsoft Azure B3 Plan with backend running on our dedicated server (see Hardware and software section). For each condition and pathway size, the visualization pipeline was executed 10 times, and the elapsed time required to fully render the pathway plot was recorded. Timing measurements captured the duration from Hyperpathway embedding to completion of plot display.

#### Data analysis and visualization

Timing results from all runs were aggregated into a single Excel file for downstream analysis. For each pathway size and execution environment, the mean time to display the plot and the corresponding standard error were calculated. Performance trends were visualized as line plots with error bars, showing display time (seconds) as a function of the number of pathways, enabling direct comparison between local and web-based execution across small to large pathway collections.

#### Computational speedup benchmarking of leaf removal and post-hoc reinsertion

To quantify the computational benefit of the leaf removal and post-hoc reinsertion pipeline (Innovations 3a–3b), embedding times were measured on the full Reactome Pathways Gene Set as a function of the proportion of leaf nodes reintroduced into the network (0–100%). Two conditions were compared: (i) the accelerated pipeline, which embeds only the pruned core network and reinserts leaves post-hoc in *O*(|${V}_{L}$ | ) time; and (ii) the original algorithm, which embeds the full network (inclusive of all leaf nodes) at each tested proportion. Benchmarks were executed on two hardware configurations: a laptop (16 GB RAM) and a workstation (256 GB RAM). For each condition, hardware, and leaf proportion, embedding + display time was recorded and speedup was computed as the ratio of original algorithm time to pipeline time at equivalent leaf proportions.

### Hardware and software

The Hyperpathway source code was executed in a workstation under Windows 10 Pro 2009 with 256 GB RAM, and an AMD Ryzen Threadripper PRO 3995WX 64-Cores 2.70 GHz. The Hyperpathway figures were generated MATLAB R2023a. The Hyperpathway web application employs a distributed client-server architecture to enable high-performance network embedding computations while maintaining responsive user interaction. The backend server (computational engine) runs on a dedicated on-premises server equipped with dual AMD EPYC 7H12 64-core processors (2.6–3.3 GHz) and 4 TB RAM, running Ubuntu 20.04 LTS. The server utilizes Python 3.11 with a multi-worker configuration (10 parallel workers) to handle concurrent hyperbolic embedding computations, including RA1 edge weighting, shortest path calculations, and singular value decomposition (SVD).

The web interface is hosted on Microsoft Azure Web App Service (B3 plan), providing a responsive Streamlit-based user interface with real-time progress tracking and interactive visualizations. The open-access web tool is freely available at https://hyperpathways.org/ with no registration required.

## Supplementary information

Supplementary Information

Supplementary Data

## Data Availability

The *demolist2* genomic data can be found at DAVID: https://david.ncifcrf.gov/tools.jsp. The metabolomic data are accessible at MetaboAnalyst 6.0 under the “Pathway Analysis” feature https://www.metaboanalyst.ca/MetaboAnalyst/upload/PathUploadView.xhtml. The lipidomic data are available from the original publication and can be downloaded from their Supplementary Data 3: https://doi.org/10.1038/s41467-023-38148-7. All these data are reported in the Supplementary Data file together with the results of their PEA.

## Code availability

The Hyperpathway source code is openly available on GitHub at: https://github.com/biomedical-cybernetics. The repository provides installation and usage guidelines to run Hyperpathway locally, including examples on how to generate the main Hyperpathway embedding figures presented in this study.
