# Supplementary material for: Hyperpathway: visualizing organization of pathway-molecule enriched interactions in omics studies via hyperbolic bipartite network embedding
Source: NPJ Syst Biol Appl. 2026 Jun 9;12:139. doi: 10.1038/s41540-026-00752-w (PMC13586325; doi:10.1038/s41540-026-00752-w)
Supplement: Supplementary file 1 [file 41540_2026_752_MOESM1_ESM.pdf]

Supplementary Information 1

Ilyes Abdelhamid[1,3], Yuchi Liu[1,3], Armel Lefebvre[1,3,5,6], Ziheng Liao[1,2], Aldo Acevedo[4], Carlo Vittorio Cannistraci[1,2,3,*]

[1]Center for Complex Network Intelligence (CCNI), Tsinghua Laboratory of Brain and Intelligence (THBI), Department of Psychological and Cognitive Sciences, Tsinghua University, Beijing, China
[2]Department of Computer Science, Tsinghua University, Beijing, China
[3]School of Biomedical Engineering, Tsinghua University, Beijing, China
[4]Biomedical Cybernetics Group, Biotechnology Center (BIOTEC), Center for Molecular and Cellular Bioengineering (CMCB), Technische Universität Dresden, Dresden, Germany
[5]Health Campus The Hague, Leiden University Medical Center, Turfmarkt 99, 2511 DP The Hague, The Netherlands
[6]Leiden Institute of Advanced Computer Science (LIACS), Leiden University, Einsteinweg 55, 2333 CC Leiden, The Netherlands

*Corresponding author e-mail: kalokagathos.agon@gmail.com

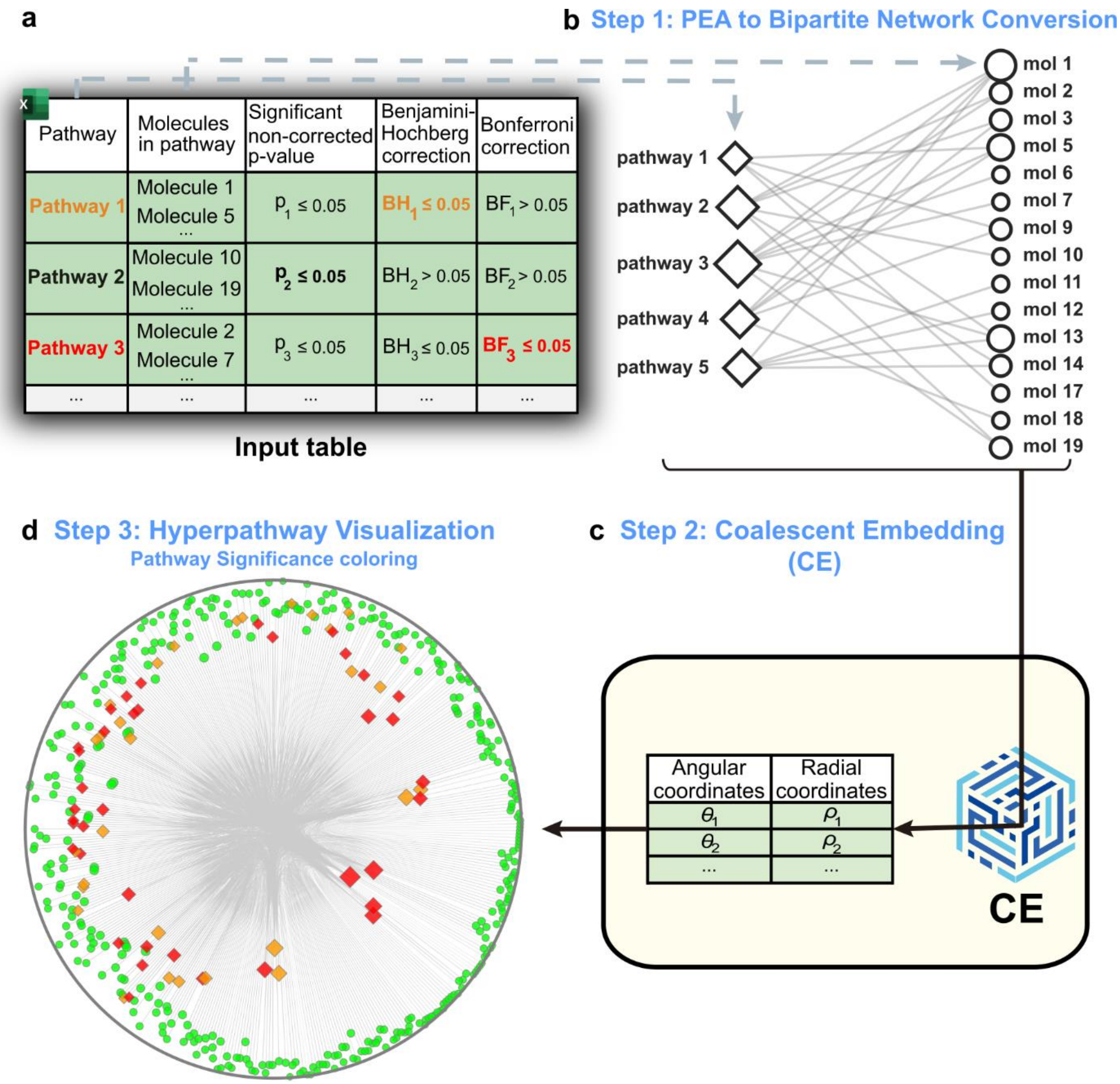

Supplementary Figure 1. Schematic overview of the Hyperpathway with pathway significance-based coloring.

a A functional enrichment table listing pathways, associated molecules, and significance values (uncorrected, BH- and Bonferroni-corrected p-values). **b** The table is converted into a bipartite graph where molecules (circles) and pathways (diamonds) are nodes, and edges represent pathway-molecule associations. **c** The bipartite graph is embedded into hyperbolic space using coalescent embedding (CE). Each node is assigned angular (θ) and radial (ρ) coordinates that reflect functional similarity and hierarchy, respectively. **d** The embedding is visualized in a hyperbolic 2D disk. Functionally related molecules and pathways co-locate into angular sectors, revealing modular structure and hierarchical organization. The colors of the embedded pathway nodes (diamonds) are assigned according to the significance of p-values in the original functional enrichment table.

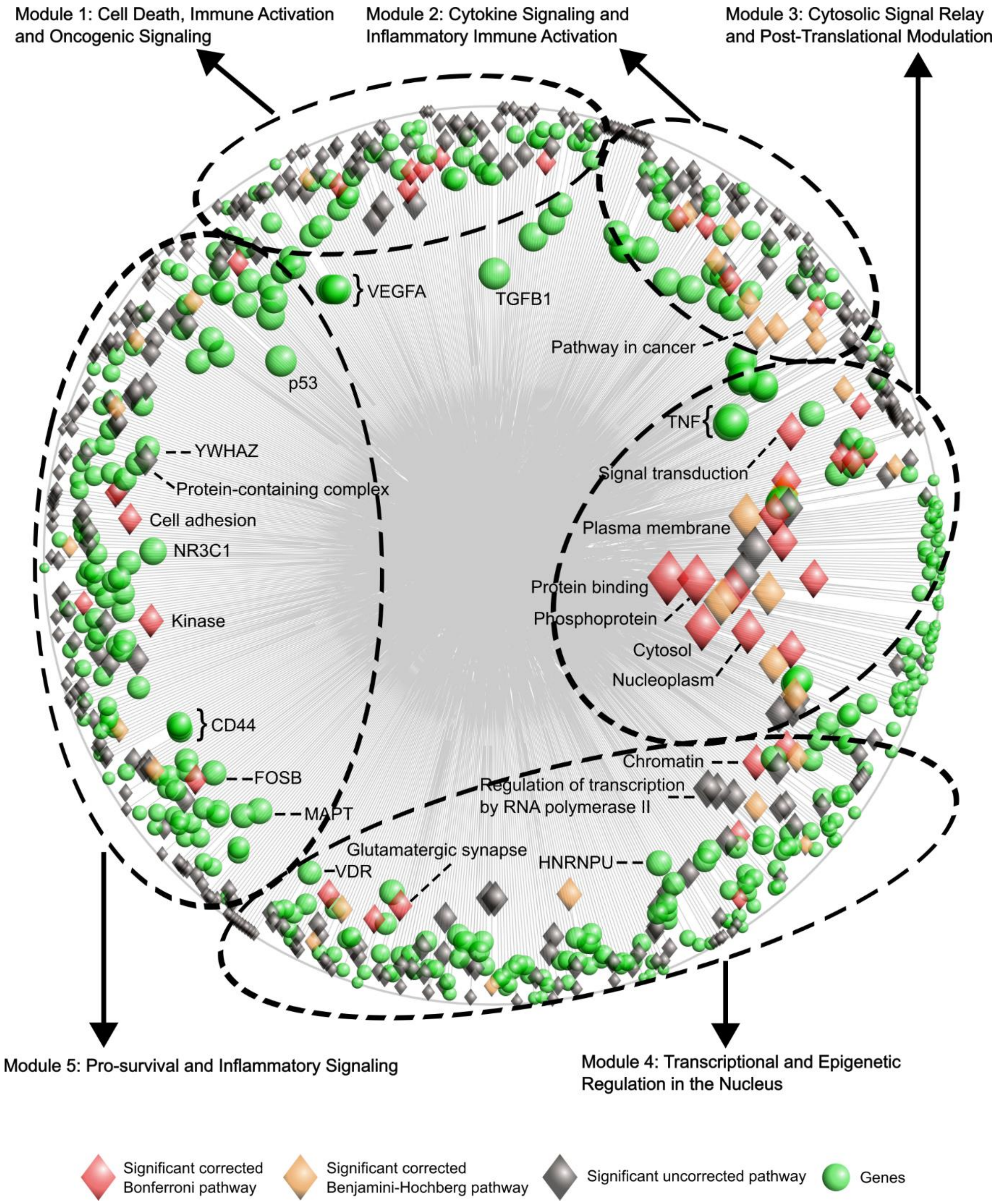

Supplementary Figure 2. Extended Hyperpathway visualization of genomic data.

Hyperpathway embedding visualization in the 2D disk of the gene-pathway interactions. Genes and enriched pathways are visualized in a hyperbolic space embedding. Nodes representing pathways are

highlighted according to their statistical significance: red indicates significant corrected Bonferroni pathways**,** orange indicates significant corrected Benjamini–Hochberg (BH) pathways, and grey indicates significant uncorrected pathways**.** Pathway nodes are scaled according to the number of enriched genes they contain, whereas genes nodes are sized by their degree (number of pathway connections) in the bipartite network. Visually identified modules are marked by dashed ellipses, highlighting the set of pathways belonging to each module. Note that for clarity only the pathways are enclosed into the dashed ellipses; associated molecules may extend beyond the ellipses.

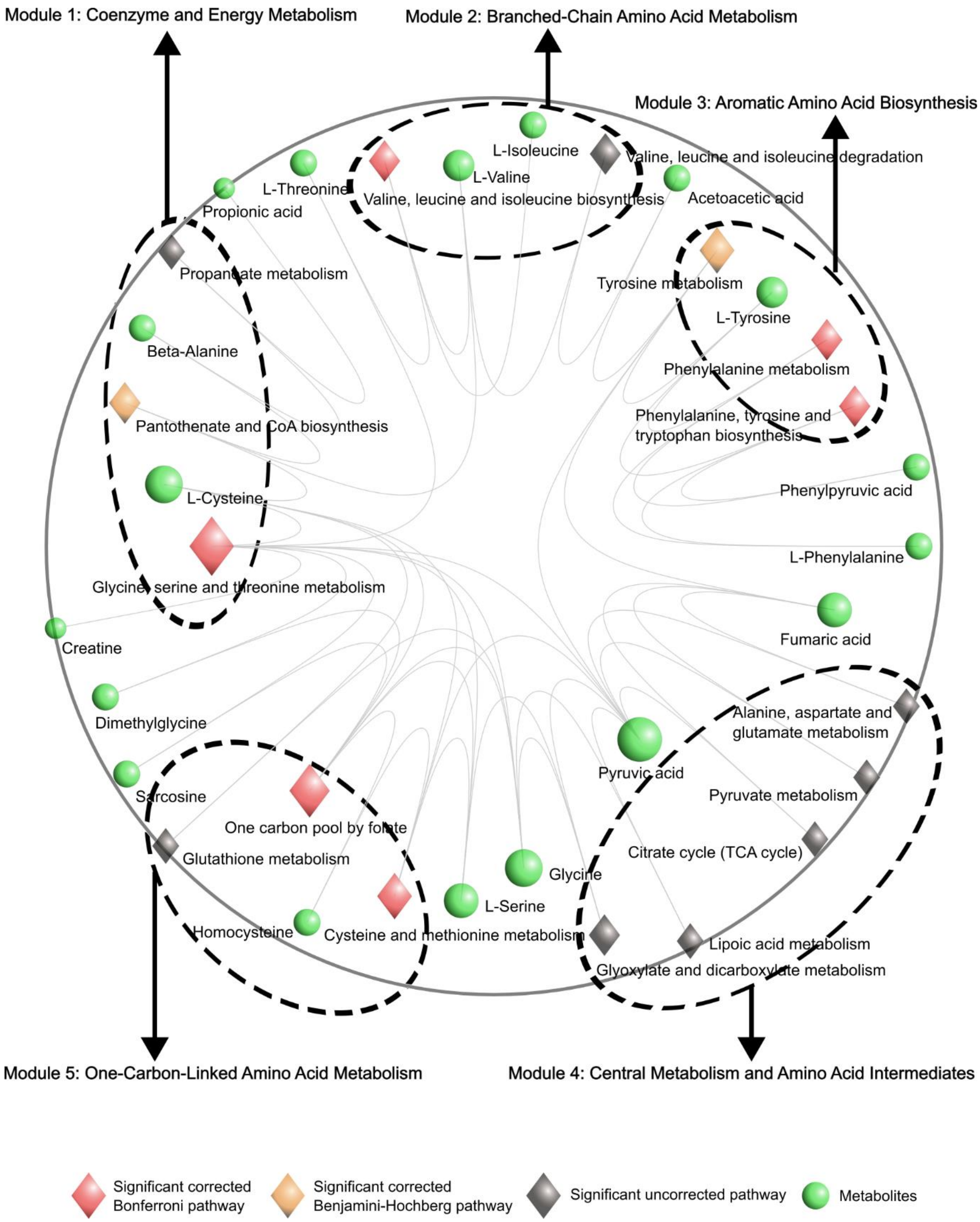

Supplementary Figure 3. Extended Hyperpathway visualization of metabolomic data.

Hyperpathway visualization in the 2D disk of the metabolite-pathway interactions. Metabolites and enriched pathways are visualized in a hyperbolic space embedding. Nodes representing pathways are highlighted according to their statistical significance: red indicates significant corrected Bonferroni

pathways**,** orange indicates significant corrected Benjamini–Hochberg (BH) pathways, and grey indicates significant uncorrected pathways**.** Pathway nodes are scaled according to the number of enriched metabolites they contain, whereas metabolites nodes are sized by their degree (number of pathway connections) in the bipartite network. Visually identified modules are marked by dashed ellipses, highlighting the set of pathways belonging to each module. Note that for clarity only the pathways are enclosed into the dashed ellipses; associated molecules may extend beyond the ellipses.

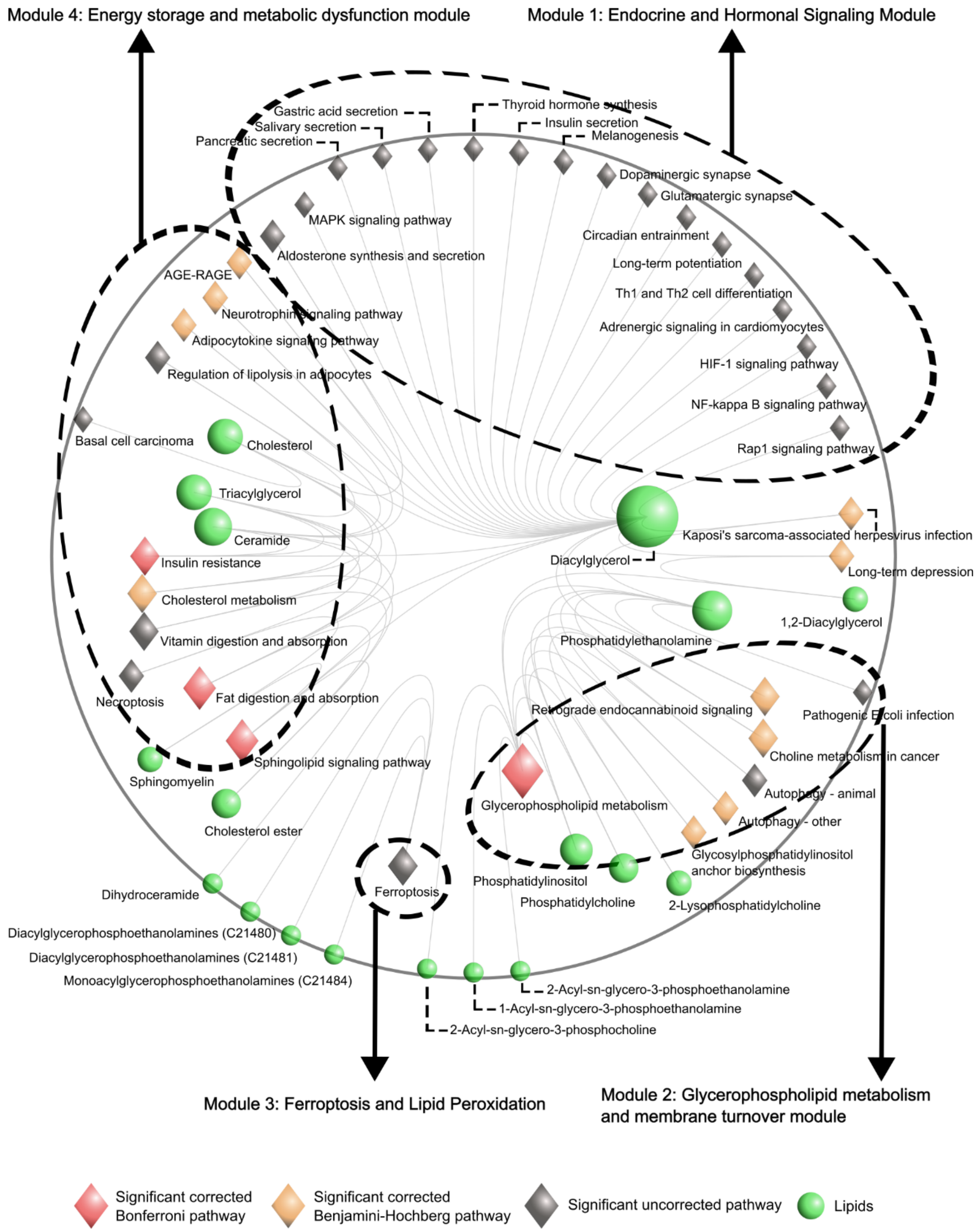

Supplementary Figure 4. Extended Hyperpathway visualization of lipidomic data.

Hyperpathway visualization in the 2D disk of the lipid-pathway interactions. Lipids and enriched pathways are visualized in a hyperbolic space embedding. Nodes representing pathways are highlighted according to their statistical significance: red indicates significant corrected Bonferroni pathways**,** orange indicates significant corrected Benjamini–Hochberg (BH) pathways, and grey indicates significant uncorrected

pathways**.** Pathway nodes are scaled according to the number of enriched lipids they contain, whereas lipids nodes are sized by their degree (number of pathway connections) in the bipartite network. Visually identified modules are marked by dashed ellipses, highlighting the set of pathways belonging to each module. Note that for clarity only the pathways are enclosed into the dashed ellipses; associated molecules may extend beyond the ellipses.

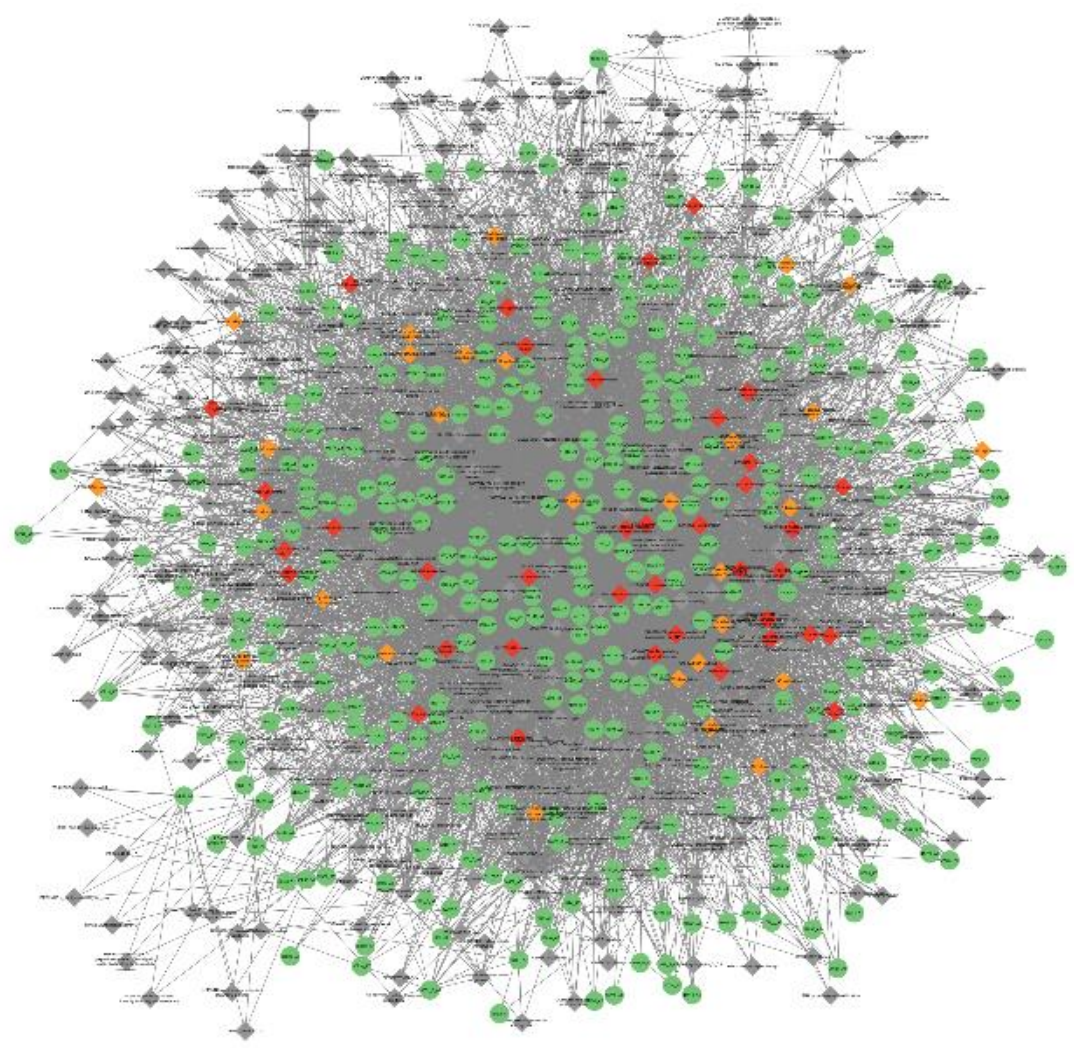

Supplementary Figure 5. Extended Cytoscape yFiles Organic Layout of genomic data.
Nodes representing pathways are highlighted according to their statistical significance: red indicates significant corrected Bonferroni pathways**,** orange indicates significant corrected Benjamini–Hochberg (BH) pathways, and grey indicates significant uncorrected pathways**.** Pathway nodes are scaled according to the number of enriched genes they contain, whereas genes nodes are sized by their degree (number of pathway connections) in the bipartite network.

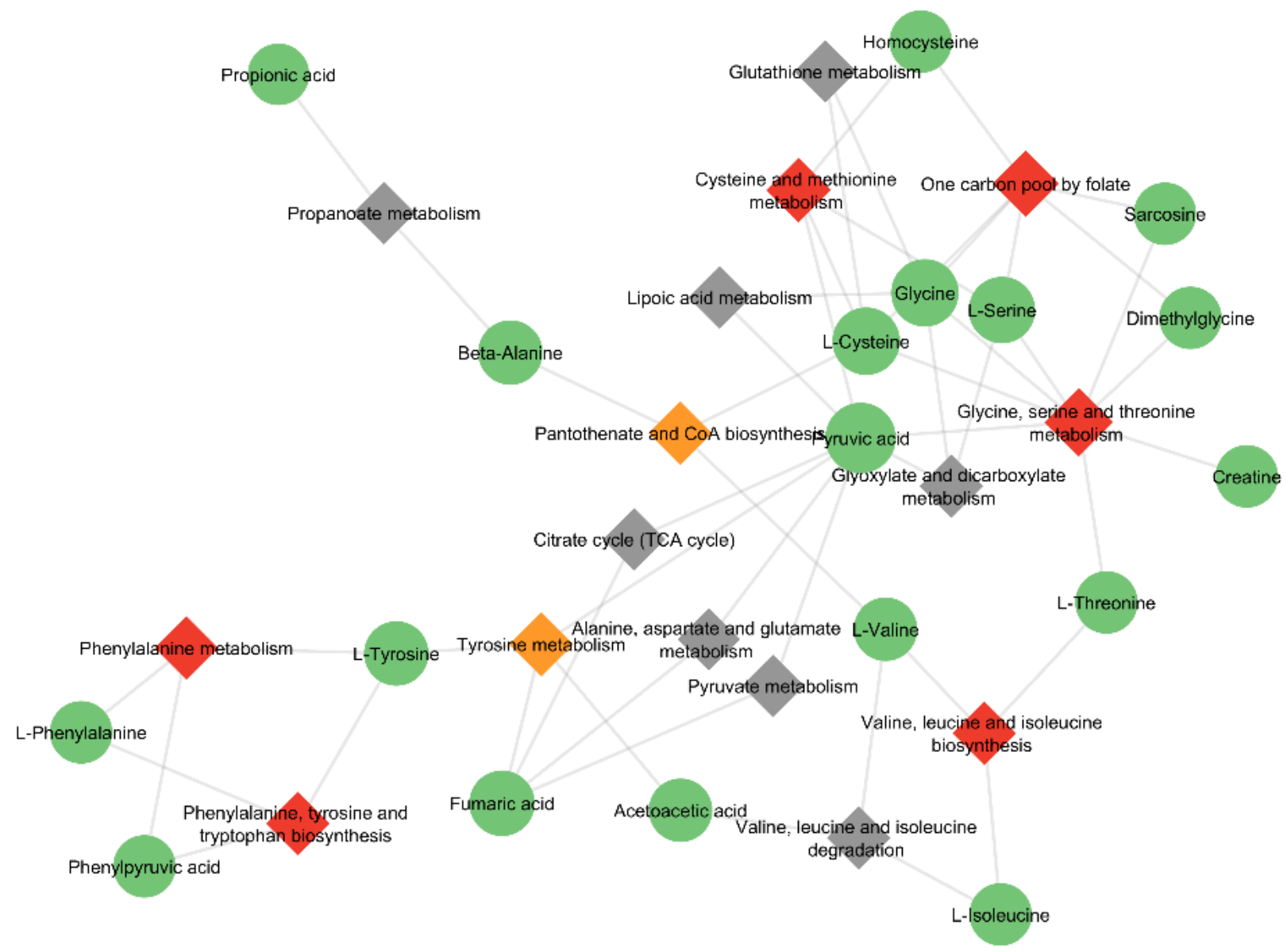

Supplementary Figure 6. Extended Cytoscape yFiles Organic Layout of metabolomic data. Nodes representing pathways are highlighted according to their statistical significance: red indicates significant corrected Bonferroni pathways**,** orange indicates significant corrected Benjamini–Hochberg (BH) pathways, and grey indicates significant uncorrected pathways**.** Pathway nodes are scaled according to the number of enriched metabolites they contain, whereas metabolites nodes are sized by their degree (number of pathway connections) in the bipartite network.

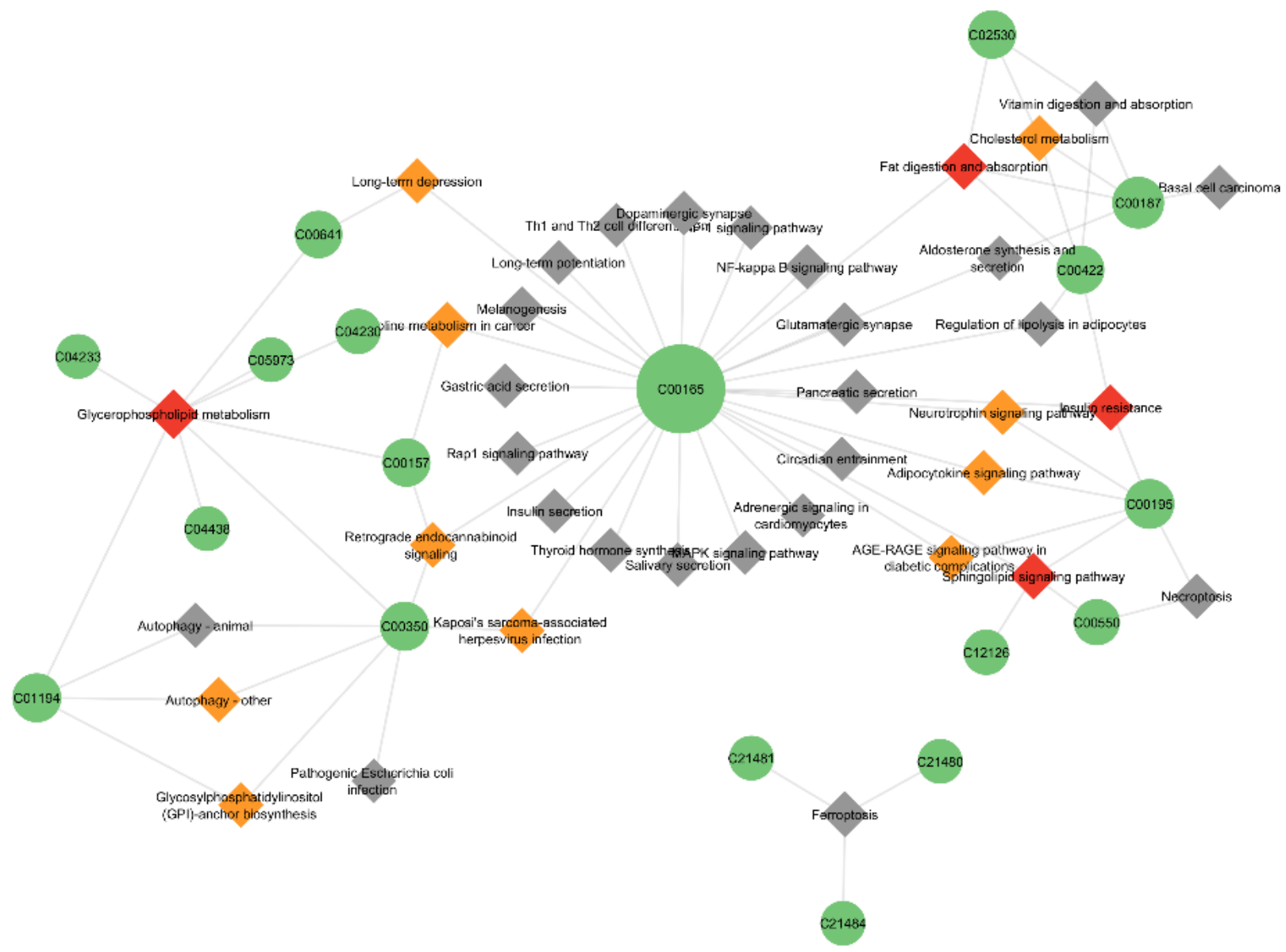

Supplementary Figure 7. Extended Cytoscape yFiles Organic Layout of lipidomic data.
Nodes representing pathways are highlighted according to their statistical significance: red indicates significant corrected Bonferroni pathways**,** orange indicates significant corrected Benjamini–Hochberg (BH) pathways, and grey indicates significant uncorrected pathways**.** Pathway nodes are scaled according to the number of enriched lipids they contain, whereas lipids nodes are sized by their degree (number of pathway connections) in the bipartite network.

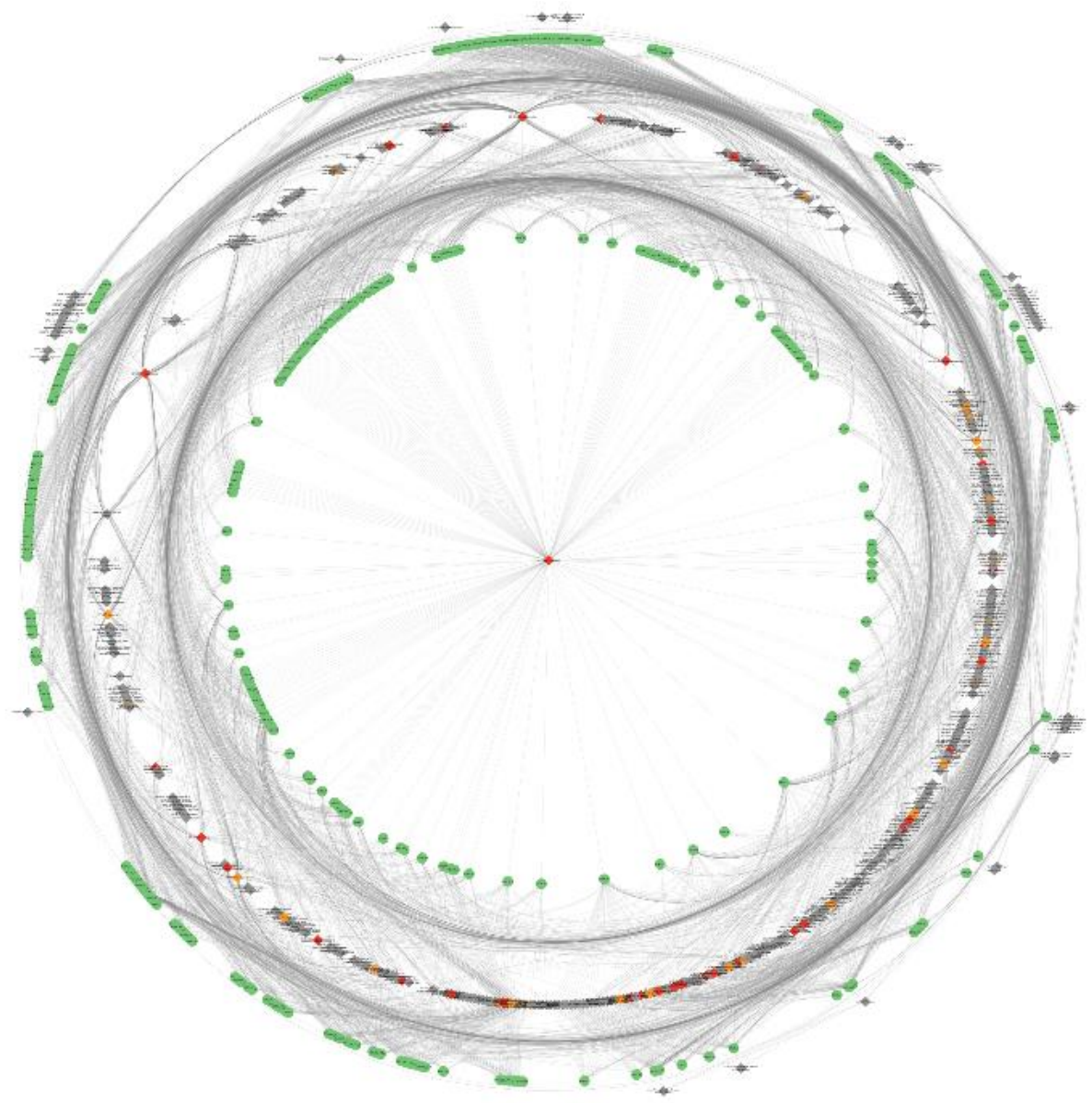

Supplementary Figure 8. Extended Cytoscape yFiles Radial Layout of genomic data.

Nodes representing pathways are highlighted according to their statistical significance: red indicates significant corrected Bonferroni pathways**,** orange indicates significant corrected Benjamini–Hochberg (BH) pathways, and grey indicates significant uncorrected pathways**.** Pathway nodes are scaled according to the number of enriched genes they contain, whereas genes nodes are sized by their degree (number of pathway connections) in the bipartite network.

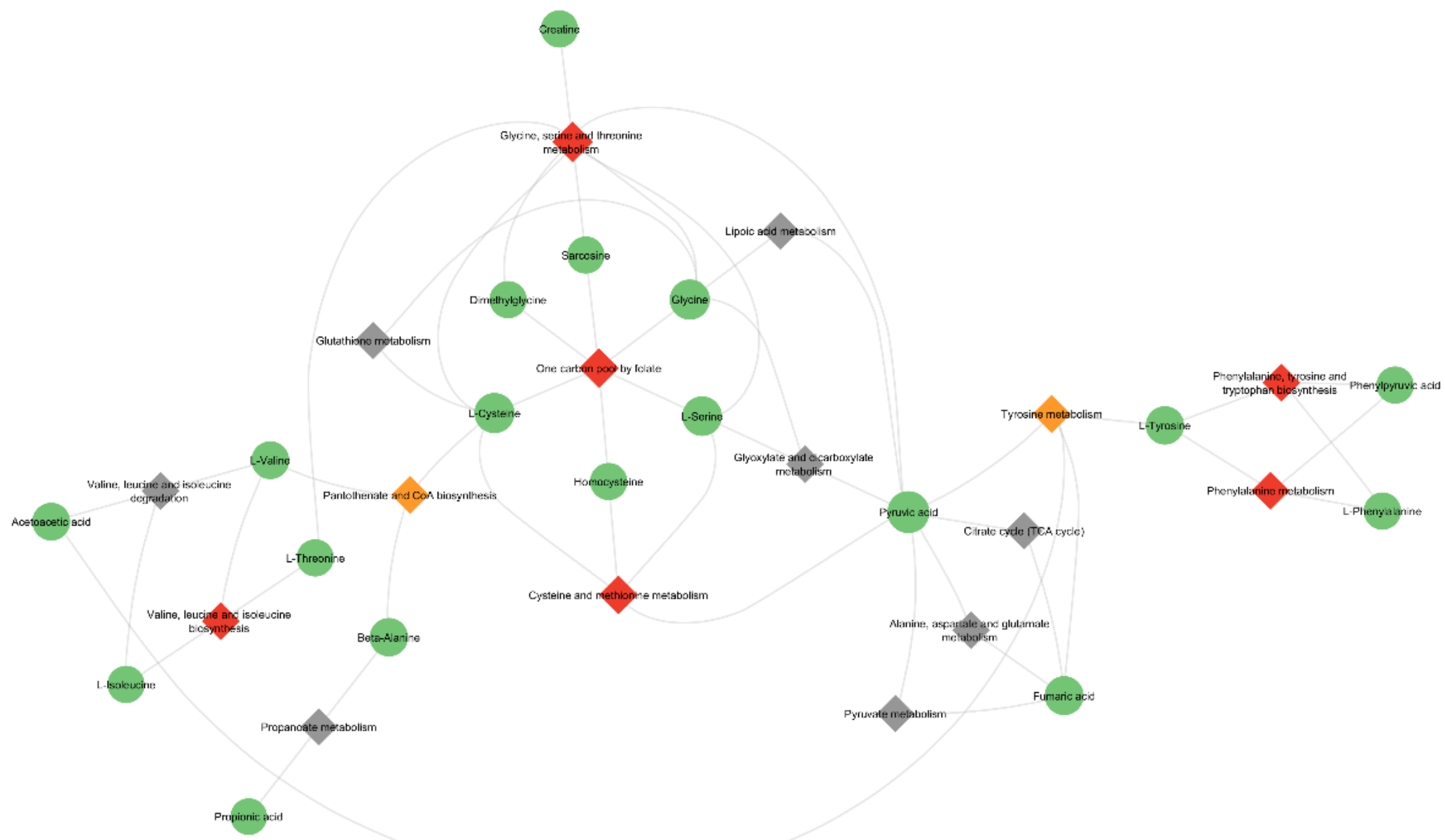

Supplementary Figure 9. Extended Cytoscape yFiles Radial Layout of metabolomic data.
Nodes representing pathways are highlighted according to their statistical significance: red indicates significant corrected Bonferroni pathways**,** orange indicates significant corrected Benjamini–Hochberg (BH) pathways, and grey indicates significant uncorrected pathways**.** Pathway nodes are scaled according to the number of enriched metabolites they contain, whereas metabolites nodes are sized by their degree (number of pathway connections) in the bipartite network.

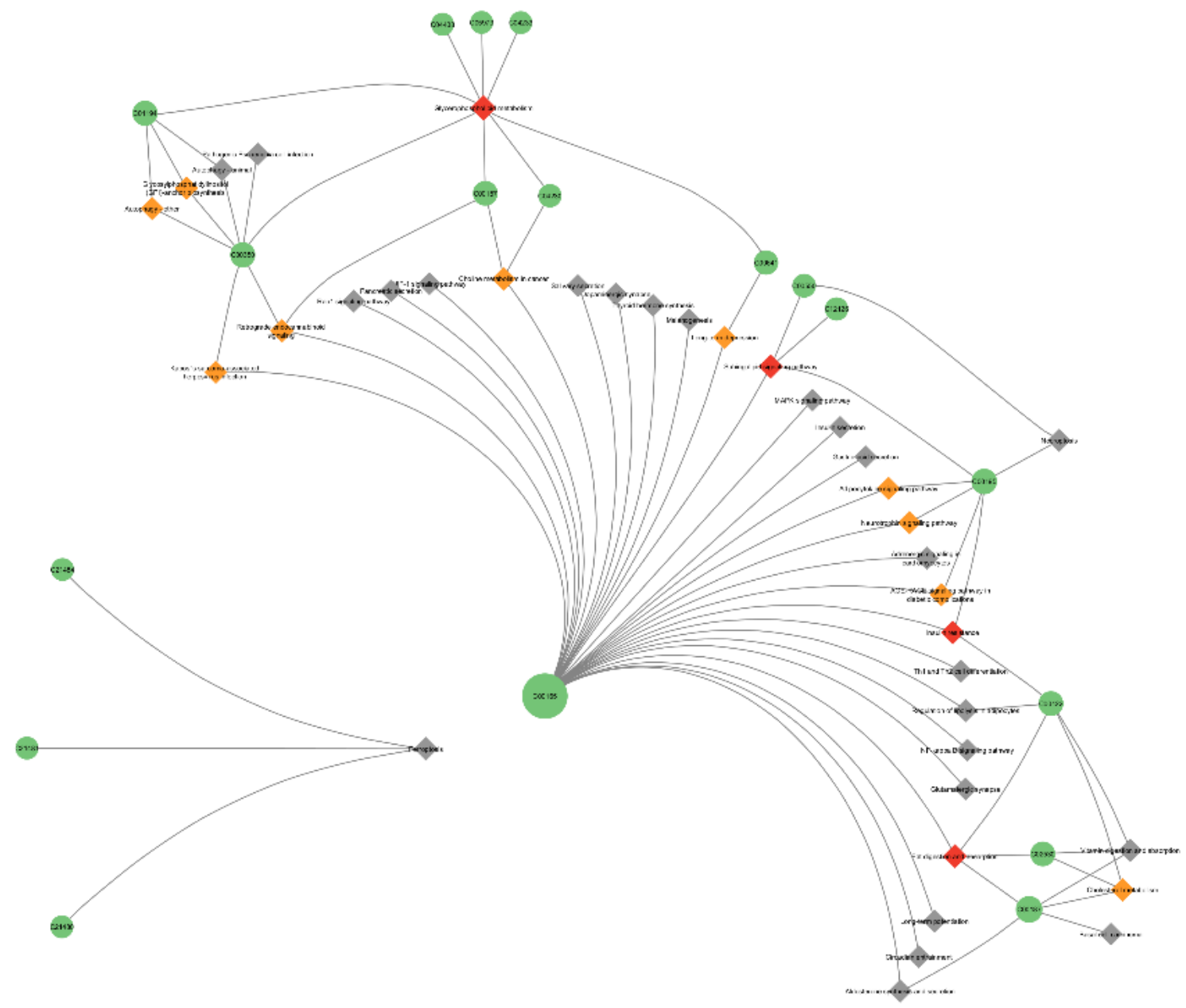

Supplementary Figure 10. Extended Cytoscape yFiles Radial Layout of lipidomic data.
Nodes representing pathways are highlighted according to their statistical significance: red indicates significant corrected Bonferroni pathways**,** orange indicates significant corrected Benjamini–Hochberg (BH) pathways, and grey indicates significant uncorrected pathways**.** Pathway nodes are scaled according to the number of enriched lipids they contain, whereas lipids nodes are sized by their degree (number of pathway connections) in the bipartite network.

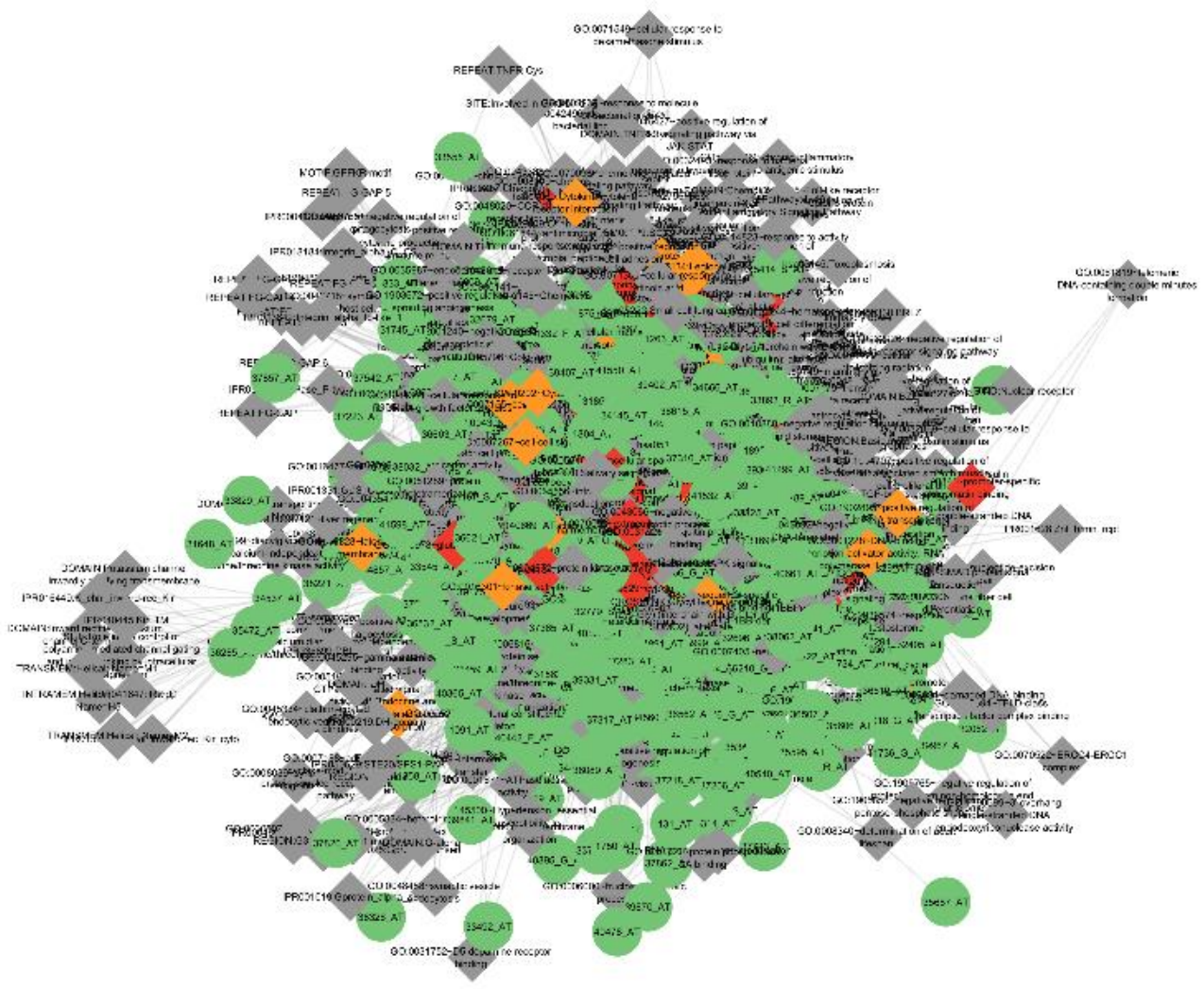

Supplementary Figure 11. Extended Cytoscape Prefuse Force Directed Layout of genomic data. Nodes representing pathways are highlighted according to their statistical significance: red indicates significant corrected Bonferroni pathways**,** orange indicates significant corrected Benjamini–Hochberg (BH) pathways, and grey indicates significant uncorrected pathways**.** Pathway nodes are scaled according to the number of enriched genes they contain, whereas genes nodes are sized by their degree (number of pathway connections) in the bipartite network.

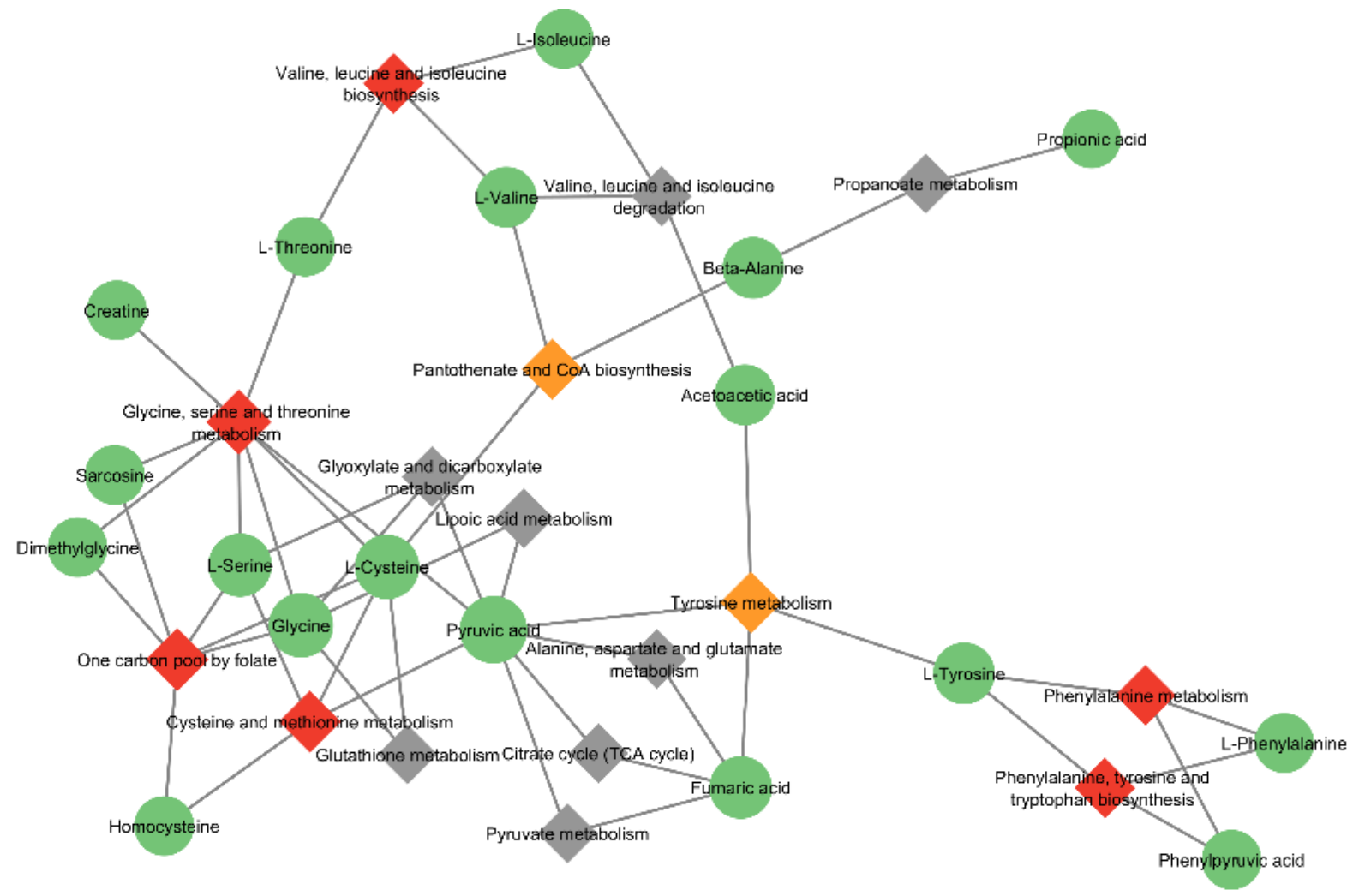

Supplementary Figure 12. Extended Cytoscape Prefuse Force Directed Layout of metabolomic data.

Nodes representing pathways are highlighted according to their statistical significance: red indicates significant corrected Bonferroni pathways**,** orange indicates significant corrected Benjamini–Hochberg (BH) pathways, and grey indicates significant uncorrected pathways**.** Pathway nodes are scaled according to the number of enriched metabolites they contain, whereas metabolites nodes are sized by their degree (number of pathway connections) in the bipartite network.

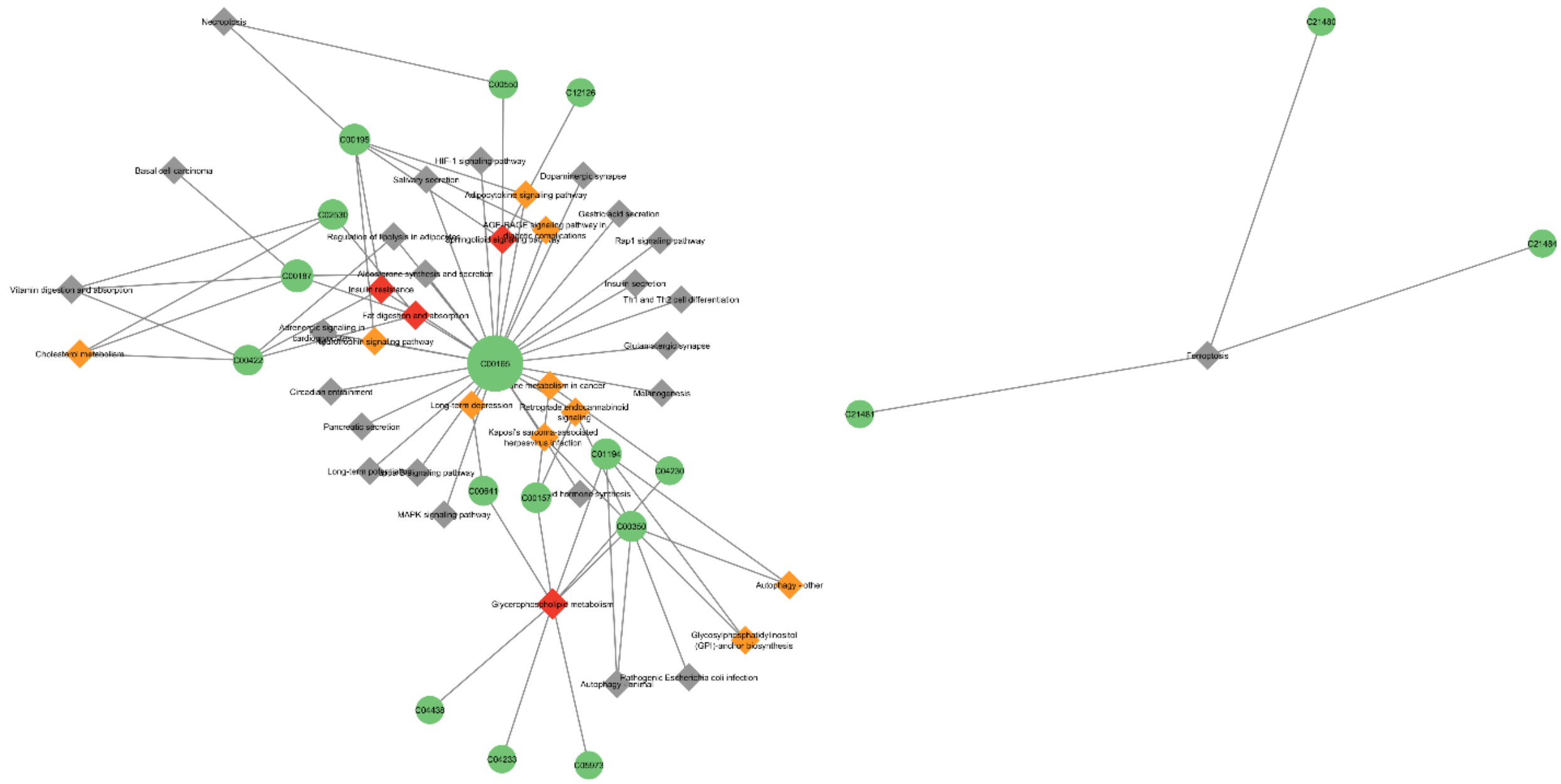

Supplementary Figure 13. Extended Cytoscape Prefuse Force Directed Layout of lipidomic data. Nodes representing pathways are highlighted according to their statistical significance: red indicates significant corrected Bonferroni pathways**,** orange indicates significant corrected Benjamini–Hochberg (BH) pathways, and grey indicates significant uncorrected pathways**.** Pathway nodes are scaled according to the number of enriched lipids they contain, whereas lipids nodes are sized by their degree (number of pathway connections) in the bipartite network.

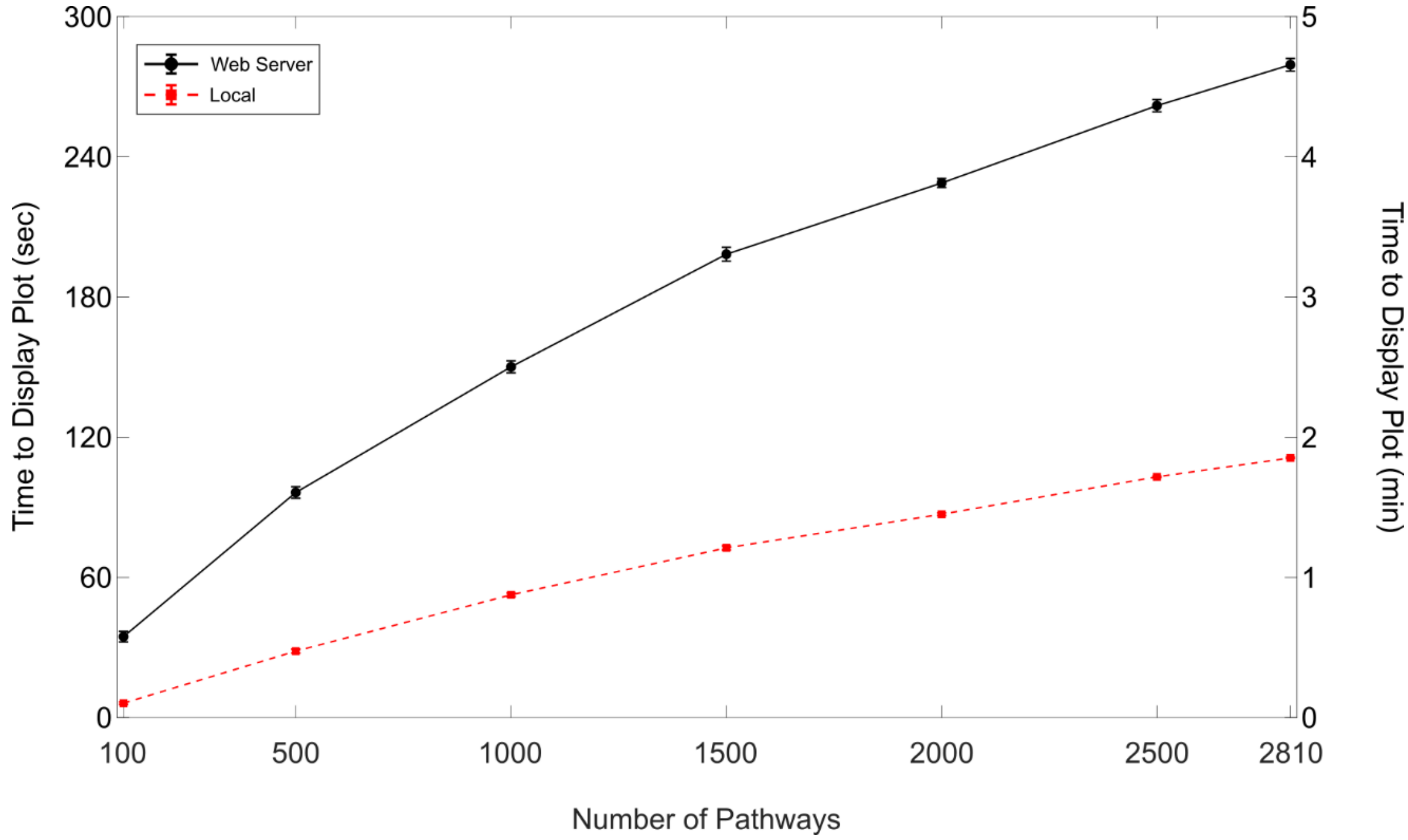

Supplementary Figure 14. Extended performance benchmarking of pathway network visualization rendered locally versus via Hyperpathway web application.

Time to display pathway network plots as a function of the number of Reactome pathways (100–2810 as complete set), comparing local execution with the Hyperpathway web application hosted on Azure Web Server (see Methods). Points show mean display time across 10 runs, with error bars indicating standard error. Black solid lines denote web-based performance, and red dashed lines denote local performance.

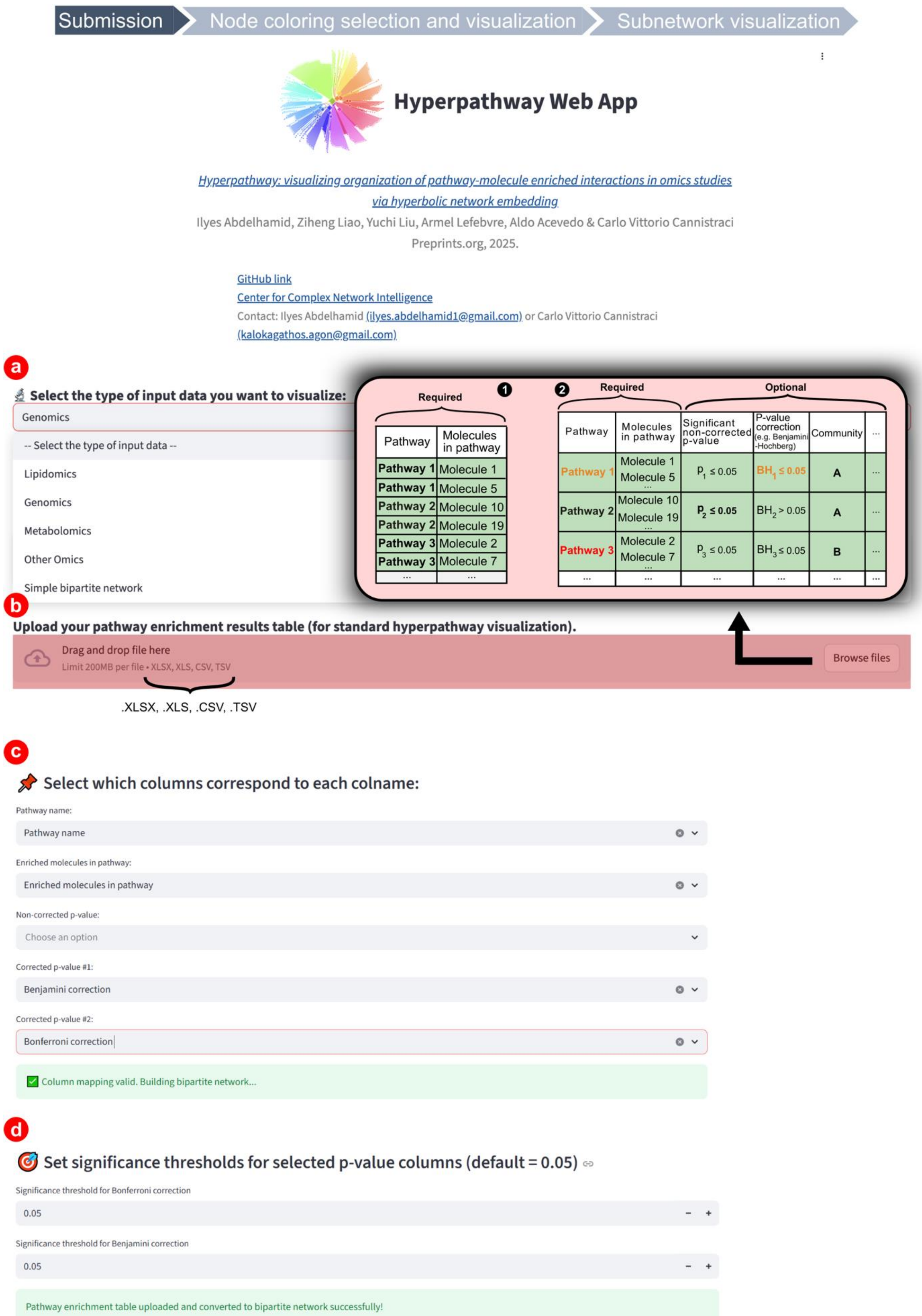

Submission
Node coloring selection and visualization
Subnetwork visualization
Hyperpathway Web App
Hyperpathway: visualizing organization of pathway-molecule enriched interactions in omics studies via hyperbolic network embedding
Ilyes Abdelhamid, Ziheng Liao, Yuchi Liu, Armel Lefebvre, Aldo Acevedo & Carlo Vittorio Cannistraci
Preprints.org, 2025.
GitHub link
Center for Complex Network Intelligence
Contact: Ilyes Abdelhamid (ilyes.abdelhamid1@gmail.com) or Carlo Vittorio Cannistraci (kalokagathos.agon@gmail.com)
a
Select the type of input data you want to visualize:
Genomics
-- Select the type of input data --
Lipidomics
Genomics
Metabolomics
Other Omics
Simple bipartite network
1
Required
Pathway
Molecules in pathway
Pathway 1
Molecule 1
Pathway 1
Molecule 5
Pathway 2
Molecule 10
Pathway 2
Molecule 19
Pathway 3
Molecule 2
Pathway 3
Molecule 7
2
Required
Optional
Pathway
Molecules in pathway
Significant non-corrected p-value
P-value correction (e.g. Benjamini-Hochberg)
Community
Pathway 1
Molecule 1
Molecule 5
$P_1 \le 0.05$
$BH_1 \le 0.05$
A
Pathway 2
Molecule 10
Molecule 19
$P_2 \le 0.05$
$BH_2 > 0.05$
A
Pathway 3
Molecule 2
Molecule 7
$P_3 \le 0.05$
$BH_3 \le 0.05$
B
b
Upload your pathway enrichment results table (for standard hyperpathway visualization).
Drag and drop file here
Limit 200MB per file • XLSX, XLS, CSV, TSV
Browse files
.XLSX, .XLS, .CSV, .TSV
c
Select which columns correspond to each colname:
Pathway name:
Pathway name
Enriched molecules in pathway:
Enriched molecules in pathway
Non-corrected p-value:
Choose an option
Corrected p-value #1:
Benjamini correction
Corrected p-value #2:
Bonferroni correction
Column mapping valid. Building bipartite network...
d
Set significance thresholds for selected p-value columns (default = 0.05)
Significance threshold for Bonferroni correction
0.05
Significance threshold for Benjamini correction
0.05
Pathway enrichment table uploaded and converted to bipartite network successfully!

Supplementary Figure 15. Pathway Enrichment Analysis (PEA) table submission user interface.

(a) Users select the type of omics data they want to visualize. This choice does not have any impact in the rest of the submission but to inform the figure legend. (b) Pathway enrichment analysis (PEA) table upload interface. The application accepts tables with a minimum of two required columns: (1) pathway name and (2) enriched molecules associated with each pathway. Statistical significance columns and others extra categories (e.g., non-corrected p-value, p-value corrections, pathway community membership, etc) are optional but enable pathway significance-based visualization and label-based one. The inset diagram illustrates the accepted table structure. Two input formats are supported: (i) one pathway-molecule pair per row, or (ii) multiple molecules per pathway in a single row with molecules separated by semicolons (;), commas (,), or pipe symbols (|) within the same cell. Accepted file formats include XLSX, XLS, CSV, and TSV with a 200MB size limit. (c) This interactive selector allows users to specify which columns in their uploaded file correspond to pathway names, enriched molecules, and optional statistical significance values (non-corrected p-values and up to two correction methods). The interface includes dropdown menus for each field, with visual confirmation (green checkmark) upon successful validation. This flexible mapping system accommodates diverse input formats from different pathway enrichment analysis tools. (d) This interactive selector allows users to set significance thresholds for the selected p-value columns. After changing the value and recomputing, the visualization will be updated. Note that users can get access to the CCNI laboratory homepage and the Hyperpathway's GitHub repository for documentation and source code.

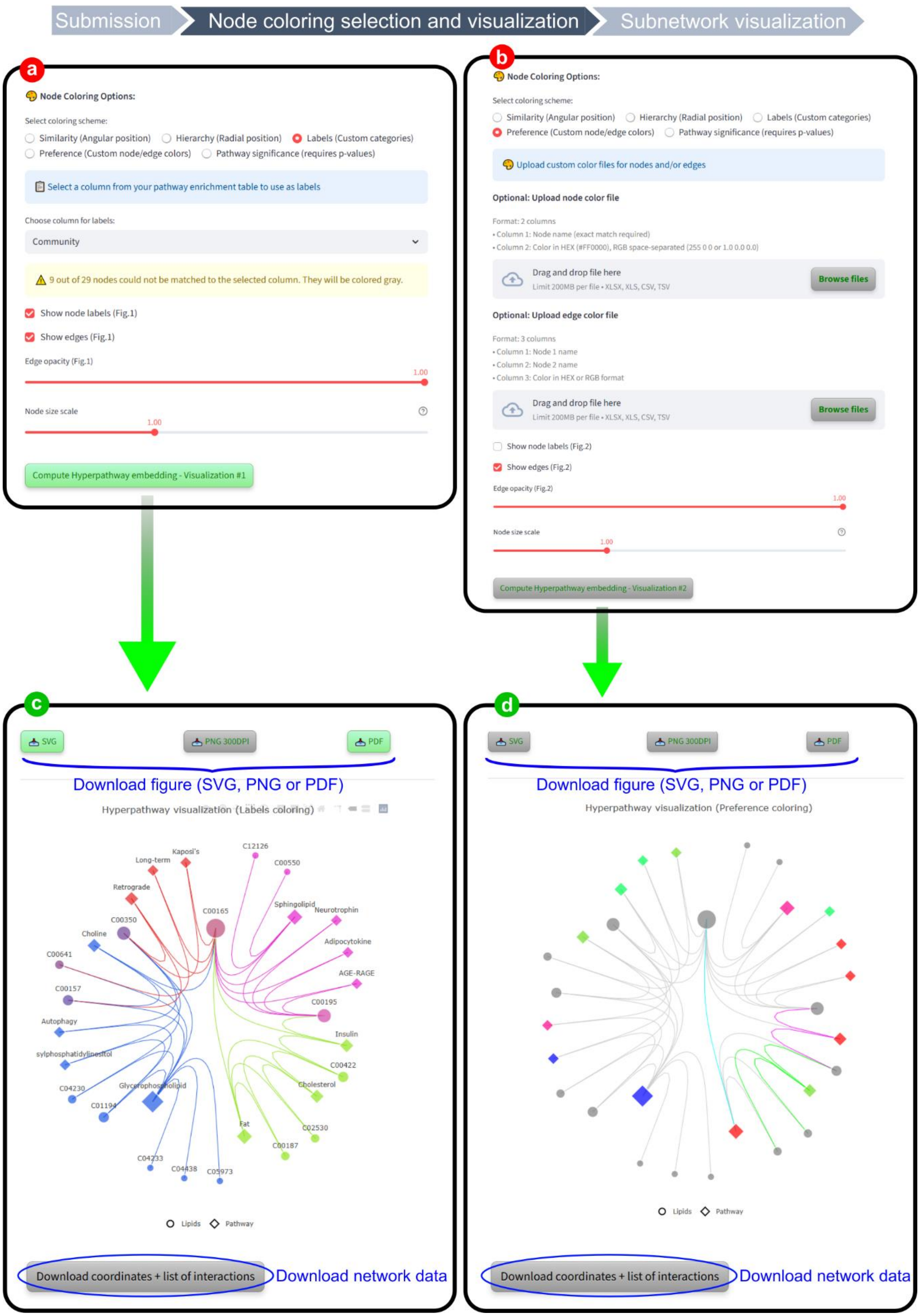

Submission
Node coloring selection and visualization
Subnetwork visualization
a
Node Coloring Options:
Select coloring scheme:
Similarity (Angular position)
Hierarchy (Radial position)
Labels (Custom categories)
Preference (Custom node/edge colors)
Pathway significance (requires p-values)
Select a column from your pathway enrichment table to use as labels
Choose column for labels:
Community
9 out of 29 nodes could not be matched to the selected column. They will be colored gray.
Show node labels (Fig.1)
Show edges (Fig.1)
Edge opacity (Fig.1)
Node size scale
Compute Hyperpathway embedding - Visualization #1
b
Node Coloring Options:
Upload custom color files for nodes and/or edges
Optional: Upload node color file
Format: 2 columns
• Column 1: Node name (exact match required)
• Column 2: Color in HEX (#FF0000), RGB space-separated (255 0 0 or 1.0 0.0 0.0)
Drag and drop file here
Limit 200MB per file • XLSX, XLS, CSV, TSV
Browse files
Optional: Upload edge color file
Format: 3 columns
• Column 1: Node 1 name
• Column 2: Node 2 name
• Column 3: Color in HEX or RGB format
Show node labels (Fig.2)
Show edges (Fig.2)
Edge opacity (Fig.2)
Compute Hyperpathway embedding - Visualization #2
c
SVG
PNG 300DPI
PDF
Download figure (SVG, PNG or PDF)
Hyperpathway visualization (Labels coloring)
Lipids
Pathway
Download coordinates + list of interactions
Download network data
d
Hyperpathway visualization (Preference coloring)

Supplementary Figure 16. Node coloring selection and visualization user interface.

The web application provides multiple node coloring options to highlight different biological features of pathway-molecule networks, and can display up to four figures on the same page (two main views and two subnetwork views). **(a)** As an example, label-based coloring mode allows users to select any column from their uploaded pathway enrichment table (e.g., "Community" shown here) to categorize and color-code pathways. Unmatched nodes are colored gray. Users can toggle node labels, edges, adjust edge opacity, and scale node sizes independently. **(b)** Preference-based coloring mode enables custom color specification via uploaded files for both nodes and edges. Users provide exact node names with corresponding colors in HEX (e.g., #FF0000) or RGB format (space-separated: 255 0 0, or 0-1 range: 1.0 0.0 0.0). Optional edge coloring follows a similar three-column format (Node 1, Node 2, Color). File size limits are 200MB for XLSX/XLS/CSV/TSV formats. **(c)** Example visualization using label-based coloring, showing distinct communities within a lipid-pathway network. Circles represent lipid molecules, diamonds represent pathways, with node sizes proportional to degree. Edge colors reflect pathway membership. **(d)** Example visualization using preference-based custom coloring, demonstrating user-defined color schemes for specific biological interpretations. Both modes support high-resolution export in SVG, PDF and PNG formats. Network topology data (node coordinates and interaction lists) can be downloaded as Excel files for downstream analysis. All visualizations maintain interactive features including zooming, panning, and node selection for subnetwork extraction.

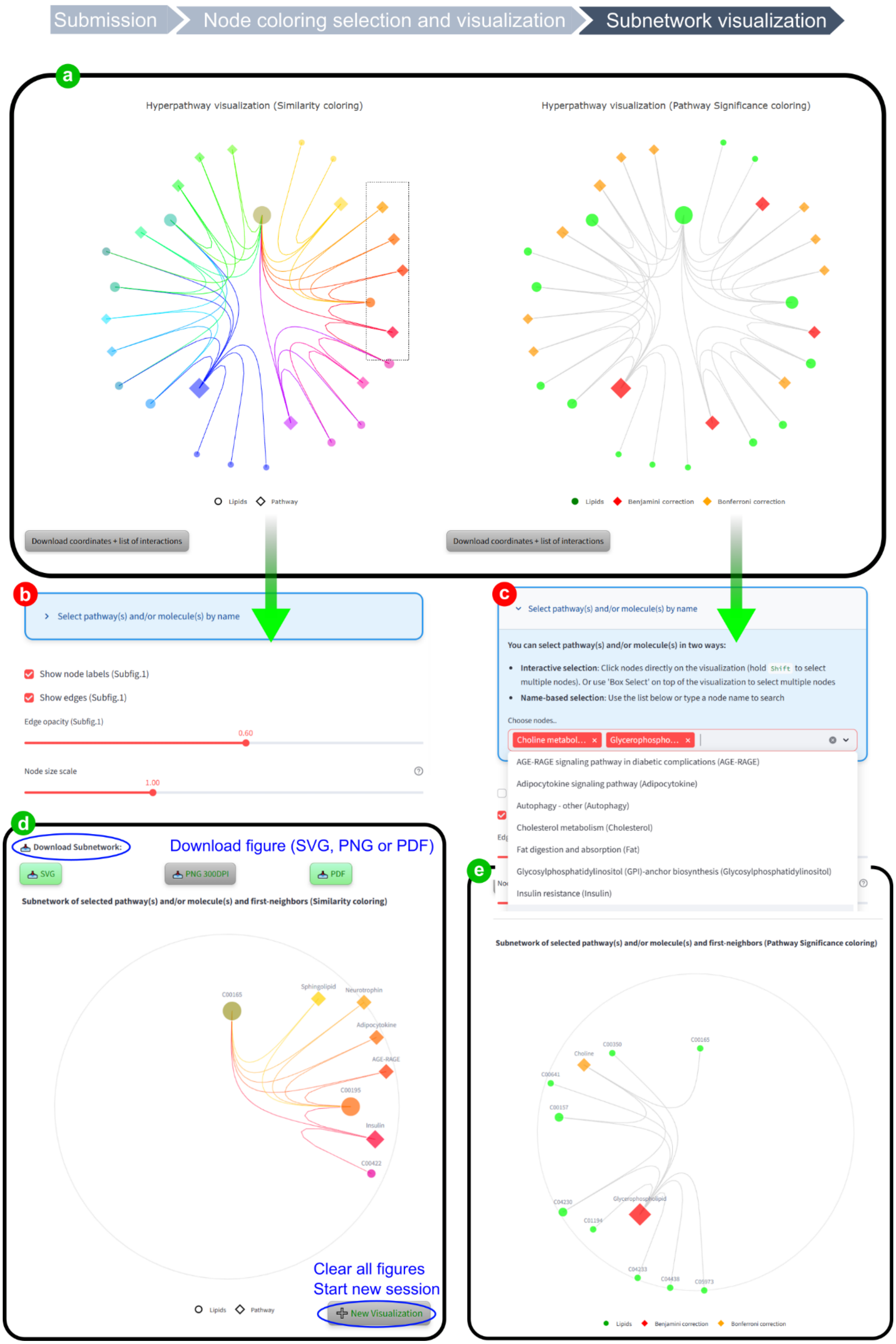

Submission
Node coloring selection and visualization
Subnetwork visualization
a
Hyperpathway visualization (Similarity coloring)
Hyperpathway visualization (Pathway Significance coloring)
Lipids
Pathway
Lipids
Benjamini correction
Bonferroni correction
Download coordinates + list of interactions
Download coordinates + list of interactions
b
Select pathway(s) and/or molecule(s) by name
Show node labels (Subfig.1)
Show edges (Subfig.1)
Edge opacity (Subfig.1)
0.60
Node size scale
1.00
c
Select pathway(s) and/or molecule(s) by name
You can select pathway(s) and/or molecule(s) in two ways:
Interactive selection: Click nodes directly on the visualization (hold Shift to select multiple nodes). Or use 'Box Select' on top of the visualization to select multiple nodes
Name-based selection: Use the list below or type a node name to search
Choose nodes..
Choline metabol...
Glycerophospho...
AGE-RAGE signaling pathway in diabetic complications (AGE-RAGE)
Adipocytokine signaling pathway (Adipocytokine)
Autophagy - other (Autophagy)
Cholesterol metabolism (Cholesterol)
Fat digestion and absorption (Fat)
Glycosylphosphatidylinositol (GPI)-anchor biosynthesis (Glycosylphosphatidylinositol)
Insulin resistance (Insulin)
d
Download Subnetwork:
Download figure (SVG, PNG or PDF)
SVG
PNG 300DPI
PDF
Subnetwork of selected pathway(s) and/or molecule(s) and first-neighbors (Similarity coloring)
C00165
Sphingolipid
Neurotrophin
Adipocytokine
AGE-RAGE
C00195
Insulin
C00422
Clear all figures
Start new session
Lipids
Pathway
New Visualization
e
Subnetwork of selected pathway(s) and/or molecule(s) and first-neighbors (Pathway Significance coloring)
C00350
C00165
Choline
C00641
C00157
C04230
Glycerophospholipid
C01194
C04233
C04438
C05973
Lipids
Benjamini correction
Bonferroni correction

Supplementary Figure 17. Subnetwork selection and visualization user interface.

(a) Main Hyperpathway visualization showing the same network rendered side-by-side using two node-coloring strategies. *Left:* similarity-based coloring, where pathway nodes are colored according to angular coordinates in hyperbolic space, reflecting functional similarity. *Right:* pathway-significance coloring, where pathway nodes are colored based on statistical significance (e.g., non-corrected and multiple-testing–corrected *p*-values), while molecule nodes are shown in green. Nodes can be selected individually or in groups directly on the visualization using click (hold Shift key to select multiple) or box-selection. **(b)** Interactive controls enabling dynamic adjustment of visual parameters, including node size scaling and edge opacity. **(c)** Alternative node-selection interface via a searchable expander panel, enabling name-based selection of pathways and/or molecules, complementary to direct graphical interaction. **(d)** Resulting subnetwork extracted from nodes selected interactively, displaying the selected pathways or molecules together with their first-neighbor connections, preserving the original coloring scheme. **(e)** Resulting subnetwork obtained from name-based selection using the expander, demonstrating equivalent subnetwork construction independent of the selection modality.

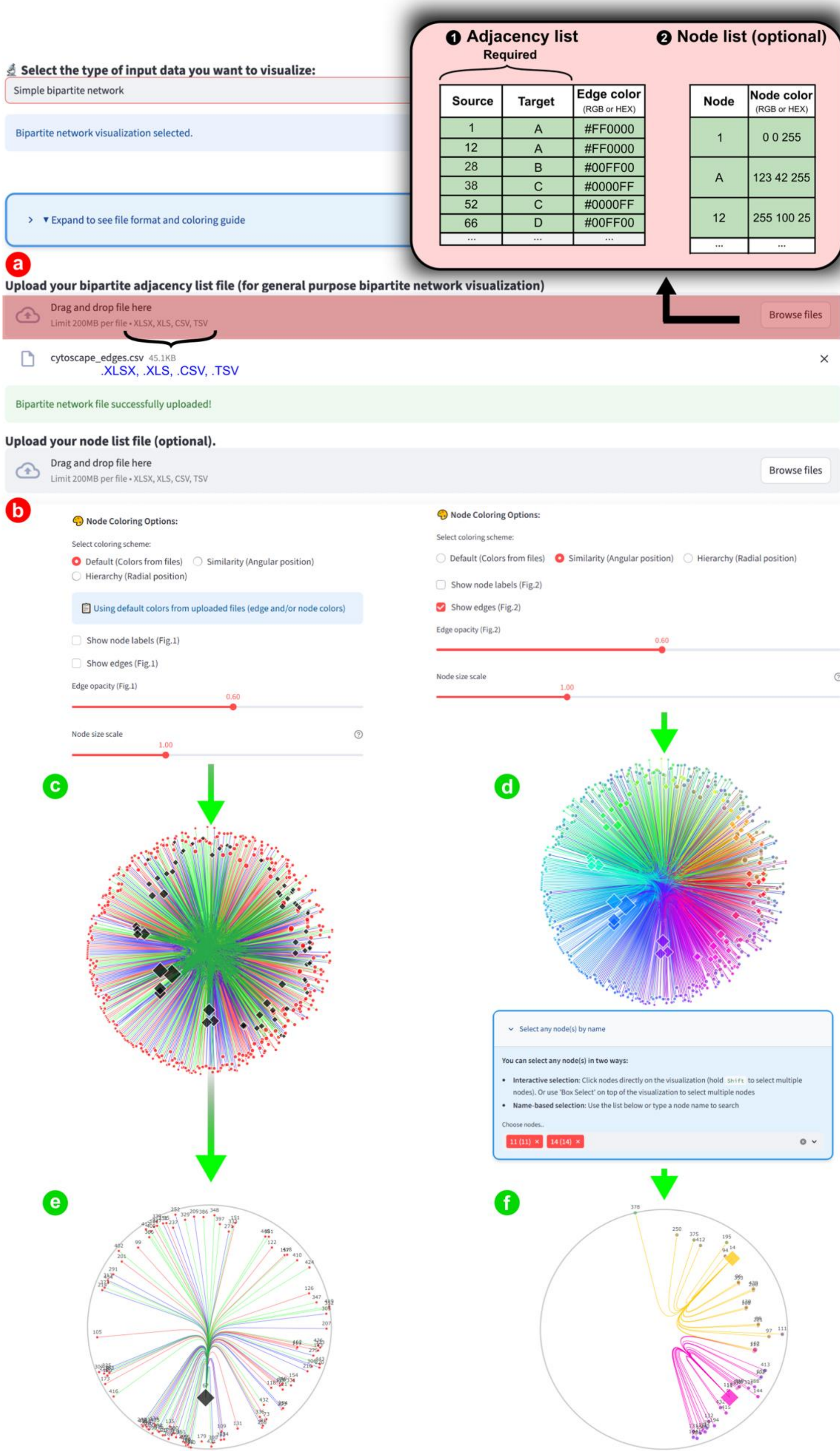

Select the type of input data you want to visualize:
Simple bipartite network
Bipartite network visualization selected.
▾ Expand to see file format and coloring guide
1 Adjacency list
Required
Source
Target
Edge color (RGB or HEX)
1 A #FF0000
12 A #FF0000
28 B #00FF00
38 C #0000FF
52 C #0000FF
66 D #00FF00
2 Node list (optional)
Node
Node color (RGB or HEX)
1 0 0 255
A 123 42 255
12 255 100 25
a
Upload your bipartite adjacency list file (for general purpose bipartite network visualization)
Drag and drop file here
Limit 200MB per file • XLSX, XLS, CSV, TSV
Browse files
cytoscape_edges.csv 45.1KB
.XLSX, .XLS, .CSV, .TSV
Bipartite network file successfully uploaded!
Upload your node list file (optional).
Drag and drop file here
Limit 200MB per file • XLSX, XLS, CSV, TSV
Browse files
b
Node Coloring Options:
Select coloring scheme:
Default (Colors from files)
Similarity (Angular position)
Hierarchy (Radial position)
Using default colors from uploaded files (edge and/or node colors)
Show node labels (Fig.1)
Show edges (Fig.1)
Edge opacity (Fig.1)
0.60
Node size scale
1.00
Node Coloring Options:
Select coloring scheme:
Default (Colors from files)
Similarity (Angular position)
Hierarchy (Radial position)
Show node labels (Fig.2)
Show edges (Fig.2)
Edge opacity (Fig.2)
0.60
Node size scale
1.00
c
d
Select any node(s) by name
You can select any node(s) in two ways:
Interactive selection: Click nodes directly on the visualization (hold shift to select multiple nodes). Or use 'Box Select' on top of the visualization to select multiple nodes
Name-based selection: Use the list below or type a node name to search
Choose nodes..
11 (11)
14 (14)
e
f

Supplementary Figure 18. Preconstructed bipartite adjacency list submission and visualization user interface.

(a) Users upload a required bipartite adjacency list (① - left table) containing source nodes (Column 1), target nodes (Column 2), and optional edge colors (Column 3) in HEX or RGB format. An optional node list file (② - right table) can be uploaded to specify custom node colors, where Column 1 contains node names (must exactly match adjacency list entries) and Column 2 contains colors in HEX or RGB format. Supported formats: XLSX, XLS, CSV, TSV. (b) Users select from available coloring schemes: Default (colors from uploaded files - nodes without colors appear black for diamonds, red for circles), Similarity (angular position-based HSV gradient reflecting topological similarity), or Hierarchy (radial position-based blue-to-red gradient by node degree). Additional controls include label visibility toggle, edge visibility toggle, edge opacity slider, and node size scaling factor. (c) Interactive hyperbolic embedding displaying the full bipartite network using the hyperbolic disk model. Edges are colored according to the uploaded adjacency list (colored edges) or default gray (no color specified). Nodes inherit colors from the uploaded node list or default colors. Node sizes scale proportionally with degree centrality. (d) Same network displayed with Similarity coloring scheme, where diamonds receive HSV gradient colors based on angular coordinates in hyperbolic space, and circles are colored as the average of their connected pathway neighbors. Edges inherit the color of their connected pathway node, creating a visually cohesive representation of network topology and functional modules. (e) Clicking on nodes in the main visualization (hold Shift for multiple selections, or use Box/Lasso tools) automatically extracts and displays first-degree neighbors in a separate subnetwork view. The subnetwork preserves the parent visualization's coloring scheme and includes inherited edge colors. (f) Alternative selection method using the expandable "Select any node(s) by name" panel. Users can search or select from a dropdown list of all node names. Selected nodes trigger automatic subnetwork extraction with first-degree neighbors. The resulting subnetwork with gradient coloring inherited from Similarity coloring mode.

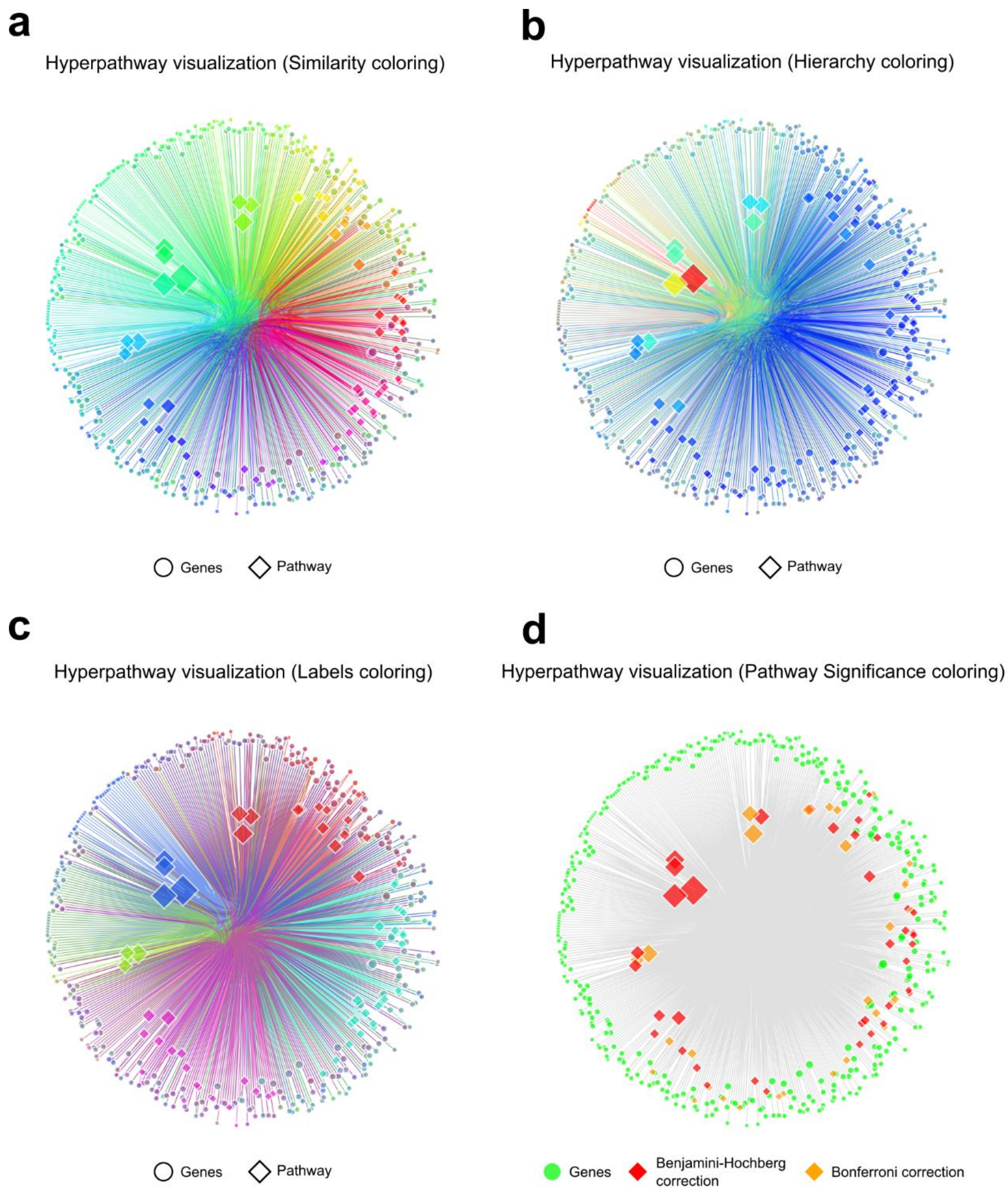

Supplementary Figure 19. All coloring modes of Hyperpathway

This figure reports all the available coloring modes of Hyperpathway.

Algorithm 1: Transforming the Pathway Enrichment Analysis (PEA) table to Bipartite network

Input: T: A five-column table displaying: (i) Pathway, (ii) enriched molecules, (iii) non-corrected p-value, (iv) Bonferroni correction, (v) BH correction).

Output: x: A (P + M) x (P + M) bipartite matrix, with P (number of unique pathways) and M (number of unique enriched molecules).

1. Read the input table in memory ***O(P)***

2. Remove duplicates and sort the unique molecules ***O(P·k + M log M)***

If on average each pathway contain k molecules, total number of molecules = P·k.

3. Initialize empty graph G ***O(1)***

4. For each row in table: ***O(P·k·M)*** (steps 4–4.b.i)

- **a. Get pathway name and associated molecule list**
- **b. For each molecule:**
  - **i. Add edge between molecule and pathway in G**

5. Matrix symmetry check ***$O((P+M)^2)$***

6. Return G

In summary, the final complexity is $O(P·k·M + M \log M + O((P+M)^2))$.

Algorithm 2: Artificial linking strategy between separated components

Input: x: Adjacency matrix (shape *NxN*).
C Number of connected components.
Output: Updated graph. Contains all original nodes and edges + additional edges (artificial links) connecting pathway nodes to molecule nodes.

1. Degree computation: $O(N^2)$
This gives the degree for each node.

2. Representative nodes per component:
For each component index i from 0 to C - 1:
- **a. Look at all nodes that belong to this component.**
- **b. Among those, find the pathway node with the most connections.**
- **c. Find the molecule node with the most connections.**
- **d. Save both nodes as representatives for that component.**

$O(C \cdot N)$

3. Build artificial links between components:
For each component index i from 0 to C - 1:
- **a. Take its chosen pathway node.**
- **b. Look at the molecule nodes from all the other components.**
- **c. Among those, find the one with the most connections.**
- **d. Link the pathway node to that highly connected molecule node.**

$O(C^2)$

4. Add Artificial Links to Graph:
- **4.1. Make a copy of the original graph.**
- **4.2. For each (pathway node, molecule node) pair that was chosen to link:**
  - **4.2.a Add a connection between the pathway and molecule node.**
- **4.3. Make sure the graph stays symmetric (undirected).**

$O(N^2)$

Overall, the time complexity is: $O(N^2 + C \cdot N + C^2)$

Algorithm 3: Leaf removal

Input: x: Graph $G = (V, E)$ as sparse adjacency matrix of the full original graph (shape $N \times N$)
node attribute vector:

- *name*: node label (pathway or molecule identifier)
- *type*: node class (pathway = 0, molecule = 1)
- *symbol*: node shape for rendering (diamond or circle)
- *color*: RGB color vector for visualization

Output: x_core: Pruned core graph G_{core}
filtered attribute vectors: *name_core*, *type_core*, *symbol_core*, *color_core*

1. Compute node degrees (X is a sparse matrix): ***O*(*N*+*E*)**

$$\deg[i] = \sum_{j=1}^{N} X_{ij}, \quad \forall i \in \{1, \cdots, N\}$$

2. Build core mask: ***O*(*N*)**

$$mask[i] = \begin{cases} True & if \deg[i] > 1 \\ False & if \deg[i] = 1 \end{cases}$$

3. Slice core adjacency matrix: $\boldsymbol{O(|V_{core} + E_{core}|)}$

$$X_{core} = X[mask, mask]$$

X is a sparse matrix so:

- Iterates over only the **|*V*_core|** rows that survive the mask
- Reads only the **|*E*_core|** non-zero entries belonging to those rows

4. Filter node attributes: $\boldsymbol{O(|V_{core}|)}$

$$a_{core} = [a[i] \mid mask[i] = True], \quad \forall\, a \in \{name, type, symbol, color\}$$

5. Return x_core, *name_core, type_core, symbol_core, color_core* ***O*(1)**

Overall, the time complexity is: ***O(N+E)***

Algorithm 4: Coalescent embedding

Input: x: Adjacency matrix.
Output: Polar coordinates of nodes (θ, r).

1. Pre-weighting rules
1.1. Repulsion–attraction rule (RA, local)

$$x_{ij}^{RA1} = \frac{d_i + d_j + d_i d_j}{1 + CN_{ij}} \text{ (if } x_{ij} = 1) \quad \text{(Eq. 1)}$$

x_{ij} value of (i, j) entry in matrix x; d_i degree of node i; d_j degree of node j; e_i external degree of node i (links neither to CN_{ij} nor to j); e_j external degree of node j (links neither to CN_{ij} nor to i); CN_{ij} common neighbors of nodes i and j.

2. Nonlinear dimension reduction

2.1 Noncentered Isomap (ncISO)

3. Angular coordinates (θ)

3.1 Circular adjustment

3.2 Equidistant adjustment

4. Radial coordinates (r)

Nodes are sorted according to descending degree and the radial coordinate of the i-th node in the set is computed according to:

$$r_i = \frac{2}{\zeta}[\beta \ln i + (1-\beta)\ln N] \quad i = 1,2,\dots,N \quad \text{(Eq. 2)}$$

N number of nodes; $\zeta = \sqrt{-K}$, we set $\zeta = 1$; K curvature of the hyperbolic space; $\beta = \frac{1}{\gamma - 1}$ popularity fading parameter; γ exponent of power-law degree distribution.

Complexity of coalescent embedding algorithms

The time complexity of the coalescent embedding algorithms can be obtained summing up the computational cost of the main steps: pre-weighting, dimension reduction, assignment of angular coordinates, and radial coordinates. All the complexities reported assume the network to be connected, therefore the number of edges E has at least the same order of complexity as the number of nodes N.

1. Pre-weighting rules

- RA: it only requires basic operations on sparse matrices, whose complexity is proportional to the number of nonzero elements, therefore $O(E)$.

2. Dimension reduction techniques

2.1. ncISO

The method first computes all pairwise shortest paths between nodes using Dijkstra's algorithm executed N times (once from each node), with complexity $O(N * (E + N) * log(N)) = O(N^2 * log(N) + N * E * log(N))$ for sparse graphs. The resulting distance matrix forms a kernel. The first principal component is discarded as it primarily captures the radial embedding direction, already independently computed from the degree distribution (power-law fitting). Therefore, k=3 singular values are computed via Singular Value Decomposition (SVD) to obtain a 2D embedding from the 2nd and 3rd components. While computing all singular values requires $O(N^3)$[1], the svds function from scipy.sparse.linalg with the PROPACK solver computes only the k largest singular values with complexity $O(kN^2)$[2], making it efficient for large networks.

3. Angular coordinates

3.1. Circular adjustment

The assignment requires the conversion from cartesian to polar coordinates. The cost is $O(N)$.

3.2. Equidistant adjustment

A sorting operation is performed, which costs $O(N * \log N)$.

4. Radial coordinates

The assignment is performed applying a given mathematical formula (see Eq. 2 above), which requires the nodes to be sorted by degree, the cost is therefore $O(N * \log N)$.

5. Conclusion

If full matrix factorization techniques are used, the coalescent embedding algorithm has a complexity of $O(N^3)$. Instead, if the truncated variants are used for ncISO, the SVD takes $O(N^2)$ since k is a small constant, therefore the algorithm has a complexity dominated by the shortest path computation, which is $O(N^2 * log(N) + N * E * log(N))$ or $O(N^2 * log(N))$ for sparse graphs where $E = O(N)$.

Algorithm 5: Post-hoc leaves reinsertion

Input: x: Sparse adjacency matrix of the full original graph (shape $N \times N$), containing all nodes including leaf nodes L.

coords: EA polar coordinates of the core (non-leaf) embedded nodes only (shape $N_{core} \times 2$), where column 0 is θ^{EA}(equidistant angular coordinate) and column 1 is r (radial coordinate).

coords_ra: RAA polar coordinates of the core (non-leaf) embedded nodes only (shape $N_{core} \times 2$), where column 0 is θ^{RAA} (repulsion-attraction angular coordinate) and column 1 is r (radial coordinate).

Output: coords_new: Polar coordinates of all N nodes in the full original graph (shape $N \times 2$), where column 0 is θ^{EA} and column 1 is r.

1. Initialize output and compute radial coordinates for all *N* nodes: **$O(N \log N)$**

Apply **Eq. 2 (Algorithm 2)** to the full graph x of size N, sorting all nodes by decreasing degree. Store in coords_new[:, 1]

2. Build global-to-embedded index map: **$O(N)$**

Identify all nodes with degree > 1 as candidate core nodes. If their count is less than N_{core} (edge case: some degree-1 nodes were retained in the core during leaf removal), promote the first remaining degree-1 nodes until exactly N_{core} slots are filled. Sort all selected indices and build the mapping: global_to_emb[g] = e, where g is the global node index and e is its position in coords.

3. Compute EA equidistant step: **$O(1)$**

$$\Delta EA = \frac{2\pi}{N_{core}}$$

4. Compute nearest neighbor in RAA space for all core nodes (vectorized) **$O(N_{core}^2)$**

Build the pairwise circular distance matrix over θ^{RAA} for all core nodes $i, j \in \{0, ..., N_{core} - 1\}$:

$$D_{ij} = \min\left(\left|\theta_i^{RAA} - \theta_j^{RAA}\right|, 2\pi - \left|\theta_i^{RAA} - \theta_j^{RAA}\right|\right)$$

Set $D_{ii} = \infty$. For each core node i, store its nearest neighbor index:

$$w_i = \underset{j \neq i}{arg\,min}\, D_{ij}$$

5. Assign EA angular coordinates to all non-leaf nodes **$O(N)$**

Sequentially copy coords[:, 0] into coords_new[i, 0] for each node i with degree > 1.

6. Group leaf nodes by parent **$O(N)$**

For each node i with degree = 1, identify parent u = sole neighbor of i in x.
Store in parent_to_leaves[u] ← [i, ...].

7. For each parent *u* and its group of *n*_leaves leaf nodes ***O*(|*L*|)**

7.1. Retrieve embedded indices ***O*(1)**

parent_emb = global_to_emb[u]; nn_emb = w_parent_emb

7.2. Determine direction of nearest neighbor *w* relative to parent *u* in EA space ***O*(1)**

Compute counter-clockwise arc:

$$d = (\theta_w^{EA} - \theta_u^{EA}) \bmod 2\pi$$

If $d = 0$: direction = +1 (default).
If $d < \pi$: direction = −1 (w is CCW from u).
If $d \geq \pi$: direction = +1 (w is CW from u).

7.3. Compute fan half-span and step ***O*(1)**

The n_{leaves} siblings must be spread in a symmetric fan around θ_{center}. Compute the ideal angular half-span to space them by exactly ΔEA:

$$ideal\_half\_span = \frac{n_{leaves} - 1}{2} \cdot \Delta_{EA}$$

Cap at π to prevent the fan from exceeding half the circle and colliding with nodes on the opposite side:

$$half\_span = min(ideal\_half\_span, \pi)$$

Compute the angular step between consecutive leaves:

$$step = \begin{cases} \Delta_{EA} & if\ n_{leaves} > 1 \\ \Delta_{EA} & if\ n_{leaves} > 1\ and\ ideal\_half\_span\ \leq \pi \quad (fan\ fits: natural\ spacing) \\ \frac{2\pi}{n_{leaves} - 1} & if\ n_{leaves} > 1\ and\ ideal\_half\_span\ > \pi \quad (fan\ capped: compressed) \end{cases}$$

7.4. Compute fan center $\boldsymbol{\theta_{center}}$ in EA space ***O*(1)**

$$\theta_{center} = \begin{cases} \left(\theta_u^{EA} + \frac{d}{2}\right) mod\ 2\pi & if\ d = -1\ (w\ is\ CCW) \\ \left(\theta_u^{EA} - \frac{2\pi - d}{2}\right) mod\ 2\pi & if\ d = 1\ (w\ is\ CW) \end{cases}$$

7.5. Assign angular coordinates to each leaf in the fan **$\boldsymbol{O(n_{leaves})}$**

For $k = 0, 1, ..., n_{leaves} - 1$:

$$\theta_{leaf_k}^{EA} = (\theta_{center} - half\_span + k \cdot step) \bmod 2\pi$$

7.6. Grid-landing check: nudge entire fan if any leaf lands on a core grid slot $O(n_{leaves})$

The EA embedding places all N_{core} non-leaf nodes at positions that are exact integer multiples of $\Delta_{EA} = \frac{2\pi}{N_{core}}$ - these are referred to as grid slots. A leaf node that is inadvertently assigned an angular coordinate coinciding with one of these grid slots would collide with an existing core node in angular space, corrupting the embedding geometry. To detect this, for each leaf in the current fan, compute its distance to the nearest grid slot:

$$\delta_{leaf} = \min\left(\theta_{leaf}\ mod\ \Delta_{EA}, \Delta_{EA} - \theta_{leaf}\ mod\ \Delta_{EA}\right)$$

If any leaf in the fan satisfies $\delta_{leaf} < 10^{-9}$ (i.e., it lands within floating-point precision of a grid slot), the entire fan is rigidly shifted by a small nudge:

$$\theta_{leaf_k} \leftarrow \left(\theta_{leaf_k} + 0.1 \cdot \Delta_{EA}\right) mod\ 2\pi \quad \forall k$$

The nudge magnitude $0.1 \cdot \Delta_{EA}$ is chosen to be small enough to preserve the fan's spatial coherence relative to its parent, yet large enough to clear any grid slot by a safe margin. The entire fan is shifted as a rigid unit — rather than individual leaves — so the relative angular spacing between leaves within the fan is preserved. Any residual duplicate angular coordinates across all nodes are subsequently resolved globally in Step 8.

8. Resolve duplicate angular coordinates across all *N* nodes $O(N \log N)$

Apply adjust_duplicates to coords_new[:, 0]: sort angles, detect runs of identical values, shift each duplicate by increments of $\delta = 10^{-7}$ rad, wrap to $[0, 2\pi)$. If duplicates remain, apply resolve_remaining_duplicates. Restore original node order.

9. Return coords_new $O(1)$

In summary, the final complexity is $O(N_{core}^2 + N \log N)$, dominated by the vectorized RAA nearest-neighbor matrix in Step 4. All leaf placement steps are $O(|L|)$, adding negligible overhead.

Algorithm 6: Plot Hyperpathway Embedding

Input: x: Adjacency matrix (shape *NxN*).
coords_native: polar coordinates from the embedding (shape *N*x2).
node_colors, names, node_shape: node metadata.
ncc: number of connected components.

Let *N*, number of nodes, and *E*, number of edges.

1. Coordinate conversion (polar to cartesian) ***O(N)***

2. Plot hyperbolic links ***O(E)***

Number of edges is E = nnz(triu(x)), with triu(x) the upper triangle of matrix x.

Each arc drawing involves geometric transformation + plotting → assume **O(1)** per edge.

3. Plot nodes ***O(N)***

Loop over *N* nodes.

In summary, the final complexity is *O(N+E)*. This is a linear time visualization algorithm relative to the size of the graph.
